# Particulate matter exposure from different heating stoves and fuels in UK homes

**DOI:** 10.1038/s41598-025-05886-1

**Published:** 2025-07-01

**Authors:** Abidemi Kuye, Prashant Kumar

**Affiliations:** 1 Global Centre for Clean Air Research (GCARE), School of Engineering, Civil and Environmental Engineering, Faculty of Engineering and Physical Sciences, University of Surrey, Guildford, GU2 7XH UK; 2 Institute for Sustainability, University of Surrey, Guildford, Surrey, GU2 7XH UK

**Keywords:** Ultrafine particles, Improved stoves, Clean solid fuel, Home heating, indoor air quality, Environmental sciences, Atmospheric dynamics

## Abstract

Traditional wood stoves emit high levels of particulate matter ≤ 2.5 μm (PM_2.5_), prompting the development of improved models to reduce emissions. However, these stoves may unintentionally increase ultrafine particle ≤ 100 nm (UFP) emissions, which can penetrate biological barriers and pose health risks. This study evaluates the impacts of four solid fuel types on indoor air quality (IAQ) in five non-smoking households in Guildford, United Kingdom, using different wood stoves (eco-design, multifuel eco-design, clear skies stage (v), and open fireplace) during winter. Indoor UFP, PM_10_, PM_2.5_, black carbon (BC), and carbon monoxide (CO) levels were measured using handheld monitors in living areas. Fuel type, room volume, stove type, and burning duration significantly influenced IAQ, exacerbated by inadequate ventilation. Open fireplaces had the highest exposure levels, followed by multifuel eco-design, eco-design, and clear skies stage (v) stoves. During burning periods, median (interquartile range) indoor pollutant concentrations were UFP: 3.6 (5.8) ×10⁴ # cm⁻³, PM_2.5_: 38.4 (65.5) µg m⁻³, PM_10_: 89.6 (89.0) µg m⁻³, and BC: 1.7 (3.6) µg m⁻³ for open fireplaces. Among improved stoves, multifuel eco-design had the highest exposure; UFP: 2.2 (4.9) ×10⁴ # cm⁻³, PM_2.5_: 14.2 (16.9) µg m⁻³, PM_10_: 37.9 (45.9) µg m⁻³, and BC: 1.5 (2.3) µg m⁻³ followed by eco-design, and clear skies stage (v) stoves. Wood briquettes produced the highest pollutant levels, followed by smokeless coal, kiln-dried wood, and seasoned wood. Burning manufactured fuels (wood briquettes) increased PM_2.5_ and UFP by 4- and 1.5-times, respectively, compared to seasoned wood. The mean CO concentration for open fireplaces was 3.1 ppm, below the World Health Organisation’s (WHO) 24-hour exposure limit guideline (3.49 ppm). Smaller rooms (< 40 m³) with longer burning durations increased exposure by 2- and 3-times compared to larger rooms (> 50 m³). Low air changes per hour (ACH) (< 1.2 h⁻¹) contributed to pollutant accumulation. Our findings indicate that residential wood burning significantly increases short-term exposure to UFPs, PM_2.5_, BC, and CO, posing potential health risks. These results underscore the need for health-focused strategies when considering wood burning for domestic heating.

**Supplementary Information:**

The online version contains supplementary material available at 10.1038/s41598-025-05886-1.

**Research highlights**

- Assessed indoor air quality in Guildford homes with four solid fuel and four stove types.
- Improved stove homes with the longest burning durations have exposure similar to open fireplace homes.
- Wood briquettes and smokeless coal increased ultrafine particle (UFP) exposure by 1.7- and 1.5-times compared with seasoned wood.
- Clearskies stage (v) stove showed lowest PM_10_ (14 µg m^−3^) and PM_2.5_ (5.2 µg m^−3^) concentration.
- All the homes have low air exchange per hour (ACH) (< 1.2 h^−1^).

## Introduction

The increasing adoption of wood stoves for household heating globally, driven by rising energy costs, has emerged as a notable source of indoor air pollution in residential environments^1–4^. There is at least one residential stove in twelve UK houses^5^. The amount of particulate matter (PM) from domestic burning in the UK increased by 35% between 2010 and 2020, accounting for 27% of the nation’s PM_2.5_ emissions^6^. In recent years, open fireplaces and wood stoves have been supplemented by more modern, energy-efficient heating systems, like eco-design and pellet stoves^7–10^.

Domestic combustion of solid fuels releases a range of hazardous pollutants, including particulate matter (PM) classified by aerodynamic diameter into ultrafine particles ≤ 100 nm (UFPs or PM_0.1_), fine particles ≤ 2.5 μm (PM_2.5_), and coarse particles between 2.5 μm and 10 μm (PM_2.5−10_), as well as the gaseous pollutants such as carbon monoxide (CO), sulphur dioxide (SO_2_), nitrogen dioxide (NO_2_), polycyclic aromatic hydrocarbons (PAH), and volatile organic compounds (VOCs)^11^. The leakage of these pollutants into the indoor environment has been linked to the stove door opening during lighting or routine refuelling^12,13^. The impact of such leakages is higher in indoor spaces because most doors and windows are closed during winter (limited ventilation), indicating lower air exchange rates. The amount and composition of these pollutants produced depend on the type of stove, fuel, and moisture content^14^.

Indoor air quality (IAQ) is a significant concern because people spend up to 90% of their time indoors, yet IAQ is not yet well understood due to its complexity^15^. Indoor air pollution caused by biomass burning results in four million premature deaths per year, more than half of which are children under the age of five^16^. Short and long-term exposure to pollution from wood-burning sources has been associated with several health effects, such as chronic respiratory diseases, heart disease, pulmonary function deficits, lung cancer, heart disease, developmental abnormalities, and harm to the lungs, kidneys, liver, nervous system, and brain^17–22^. A recent study on the association between exposure to indoor smoke from fireplaces, wood stoves, and lung cancer among 50,226 non-smoking women in the United States showed that increased use of woodstoves and fireplaces was linked to a 70% greater risk of lung cancer^20^.

Table S1 summarises the relevant previous studies that have used wood stoves as a significant source of indoor UFP^23–25^ PM_2.5_^13,26–32^, PM_10_^33^, PAHs^34^ and outdoor emissions^33,35–37^. Several studies have been carried out to assess the impact of stove types (open fireplace and closed wood-burning stoves) on IAQ. For example, Vicente, et al.^38^ studied particle emissions from one open fire and one wood stove in a laboratory setting.

They showed that the PM_10_ concentrations increased 12-times for the open fire and 2-times for the wood stove during the burning periods. In addition, it was also reported that eco-design stoves can reduce PM_2.5_ emissions by 9-times compared to an open fireplace^39^. Thus, replacing old stoves with improved eco-design stoves is one intervention to reduce pollutant concentrations in homes heated with wood stoves.

Numerous studies have been carried out to assess the effect of improved heating stoves on indoor and outdoor air quality by replacing old stoves with improved stoves. They report inconsistent results. While some studies reported no significant improvement in the IAQ^40–42^ due to improved heating stoves, others have reported lower levels of indoor and outdoor PM_2.5_^29,30,43,44^. For instance, Frasca, et al.^45^ studied the influence of improved wood-burning stoves on IAQ and found high concentrations of Mn and Cu in the indoor air sample compared with outdoor air. Likewise, Chakraborty, et al.^13^ studied improved stoves and showed high exposure to PM_2.5_ and PM_1_ concentrations in a short period under normal operating conditions. They found that the peak hourly averages for PM_2.5_ and PM_1_ were 123.9% and 133.09% higher, respectively, compared to daily averages. This increase was not due to outdoor infiltration.

UFPs, compared to PM_2.5_ and PM_10,_ have drawn significant attention in recent years because of their larger surface area, distinct chemical composition, higher alveolar deposition fraction, and propensity to translocate into the human circulatory system^46–49^. Additionally, they can cause inflammation and penetrate through cell membranes^50^and accumulate in secondary organs^51^ including brain tissue^52^. Several studies have analysed the UFP emissions from daily indoor activities such as cooking, burning candles, hair drying, ironing, smoking, cleaning, electric heating, vacuuming, and spraying^53^. These exposures can often be higher than ambient concentrations^54^. Few studies have shown that using woodburning stoves is a source of indoor UFPs^23–25,55,56^ and even fewer tested various clean fuels with improved stoves and open fireplaces for emission reduction in the laboratory^57–59^. However, the scientific literature concerning UFP emission from domestic solid fuel combustion and personal UFP exposure under real-world conditions is insufficient. To fill this gap, we carried out real-world monitoring in homes with improved wood-burning stoves and open fireplaces to reveal the reality of exposure to indoor concentration to unaware users and propose measures to mitigate their exposure. To our knowledge, no field study in the literature has evaluated the effects of burning commercially available clean fuels in a range of modern improved stoves (and compared them to open fireplaces) in real-world household settings.

This study aims to fill this research gap, with an overall objective to measure UFPs, PM_1_, PM_2.5_, PM_10_, BC along with CO and CO_2_ concentrations in residential homes with improved stoves and an open fireplace. In each home, four types of clean fuels were used for burning to quantify air pollution exposure. The specific objectives were to (i) assess the indoor air pollutant concentrations from the different stove fuels, (ii) characterise the impact of different properties such as room volume, fuel types, ventilation, and burning duration on indoor pollutant concentration, (iii) thermal comfort across all homes, (iv) ACH and ventilation rate (v) UFP hazard ratio.

## Results and discussion

### Home and burning characteristics

The qualitative and quantitative information in building and occupant surveys (Supplementary Information, SI, Table S2) were assessed to understand the variation among the studied homes. The monitored homes were classified according to stove types, fuel types, room volume, and burning duration to identify the factors influencing the aerosol concentration within the different indoor microenvironments across the studied homes for each section.

Across all the investigated homes, at least two persons, on average, occupied the living/dining area while burning solid fuels for 415 ± 230 min day^−1^. The woodburning stove was installed in the living room in HO1, HO3, and HO5, while HO2 and HO4 have woodburning stoves installed in the dining area. The average room volume was 64.1 m^3^ which ranged between 39.3 and 88.5 m^3^. All the homes are naturally ventilated through doors and windows. Due to winter, most windows in all homes were closed, and the door was only opened during use, except HO1, where living room windows were opened during cooking in the kitchen. The ratio of openable areas (windows + door) to the room floor area across the home ranges from 0.29 to 0.71. HO5 had the lowest ratio of 0.29, followed by HO2, HO1, HO3, and HO4, which had the highest ratio (0.71) due to additional roof lights or skylights. Burning duration is an important factor influencing the concentration of pollutants and the exposure level produced in the indoor environment. Unlike other homes, the burning duration in HO2 is higher compared to HO1, HO3, HO4, and HO5 because the wood burning was the primary heating source in HO2, and the burning was done all day. In addition, HO2 had a permanent occupant who works from home, unlike other homes where the wood burning is turned on in the morning or evening during most working days.

### Thermal comfort across homes

**Fig. 1:**
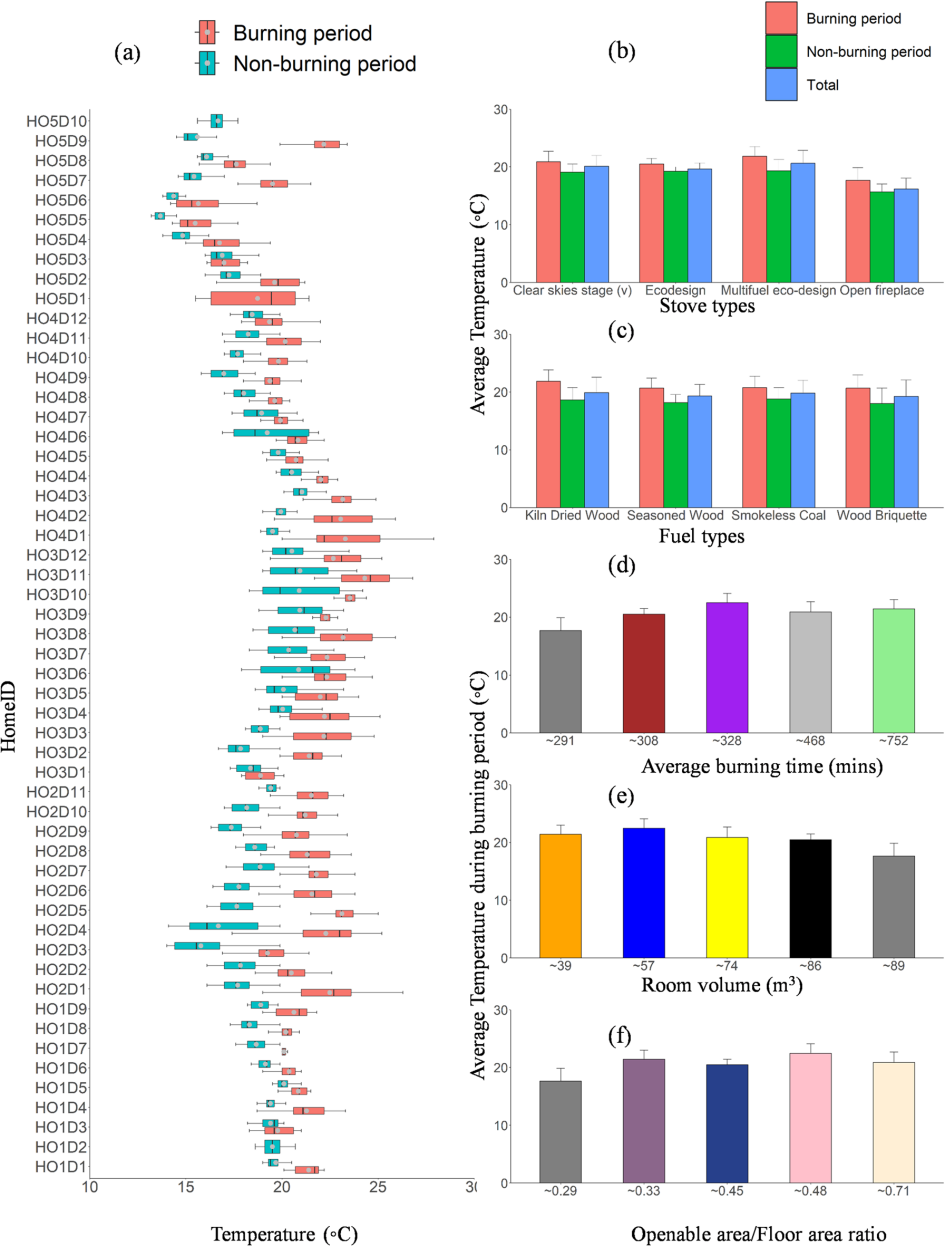
(**a**) Box plots of minute average temperature measured for all homes as denoted by home code. The plot includes the median (shown by horizontal bars), the 25th and 75th percentiles (shown by the bottom and top edge of the boxes), and minimum and maximum values (shown by the bottom and the top). The mean temperature for the entire monitoring period in each home is categorised based on (**b**) stove type, (**c**) fuel type, (**d**) average burning time, (**e**) room volume, and (**f**) openable area-to-floor area ratio. Error bars indicate the standard deviation of the average values, with only positive standard deviation values shown to maintain figure cla.

**Fig. 2:**
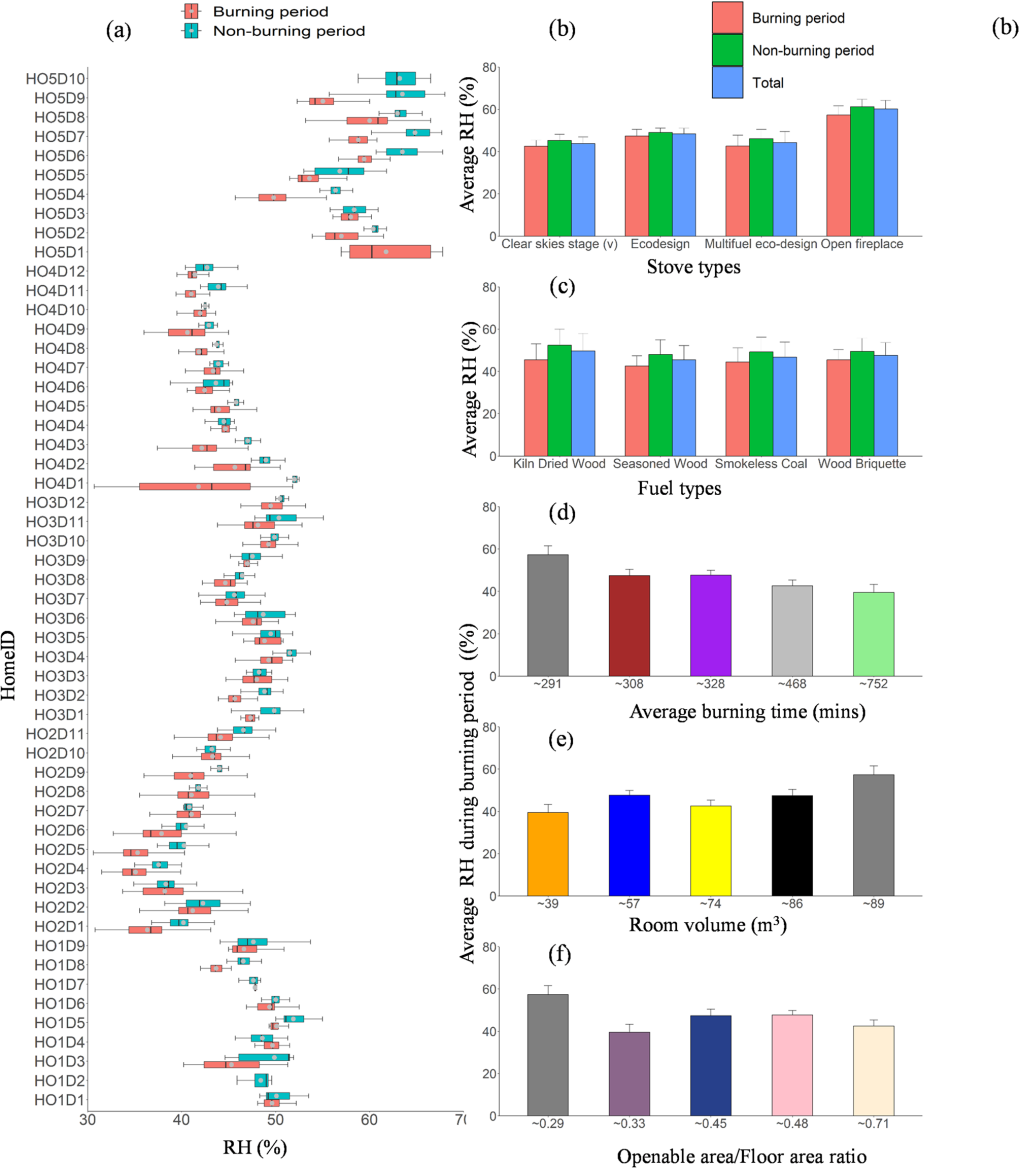
(**a**) Box plots of relative humidity (RH) measured for all homes as denoted by home code. The plot includes the median (shown by horizontal bars), the 25th and 75th percentiles (shown by the bottom and top edge of the boxes), and minimum and maximum values (shown by the bottom and the top). The average RH for the entire monitoring period in each home is categorised based on (**b**) stove type, (**c**) fuel type, (**d**) average burning time, (**e**) room volume, and (**f**) openable area-to-floor area ratio. Error bars indicate the standard deviation of the average values, with only positive standard deviation values shown to maintain figure clarity.

Figures 1 and 2 show the average temperature and relative humidity (RH) across the studied homes. The average burning duration in our study ranged from 300 to 800 min day^−1^ during the winter season. We assessed the variations in temperature and humidity across the studied homes because heat dissipation from the wood-burning stove cannot be regulated mechanically compared to other heating sources. Therefore, it is important to understand indoor thermal comfort for occupants’ health in each home and the best fuel that gives the highest heat. According to the American Society of Heating, Refrigerating, and Air-Conditioning Engineers, the recommended indoor thermal comfort for temperature and RH ranges between 21 and 23 °C and 40–60%, respectively^60^. Table S3 lists the descriptive data of the room RH and temperatures during the monitoring campaign. The overall average RH across all homes was 55 ± 8%; it was 40 ± 6.5% during the burning period (Fig. 2a), while the average temperature varies from 20 to 25 °C across all homes (Fig. 1a). On average, the room temperature rose by 5–9 °C within the burning period, while the RH reduced, as expected, from 45 − 32%.

Furthermore, we examined the average RH and temperature during the burning period based on stove type, fuel type, average burning time, room volume, and openable area/Floor area ratio (Figs. 1 and 2). Based on the average burning time, the average RH increases as the average burning time reduces (Fig. 2d). During the burning period, the highest average RH was found for HO5 (57%), followed by HO3 (48%), HO1 (47%), HO4 (42%), and HO2 (39%). This observation shows that four homes, except HO2 did not comply with the ASHRAE guidelines for thermal comfort^60^. The RH was high in HO5 due to the stove type (open fireplace) (Fig. 2b), which is known for low combustion efficiency, lower heat dissipation, and large room volume (Fig. 2e). The low RH (< 40%) in HO2 could be attributed to stove type (improved multifuel stove), high average burning time, and very small room volume (Fig. 2e). In contrast, the average temperature increases with an increase in average burning time (Fig. 1d). During the burning period, the highest average temperature was found for HO3 (22.5 °C), followed by HO2 (21.5 °C), HO4 (21 °C), HO1 (20.5 °C), and HO5 (17.7 °C). Furthermore, the indoor room RH was grouped according to the fuel type (Fig. 2c). Four fuel types were used for the experiment across four homes except HO1, where three were used due to the stove type (wood only). Based on fuel types across all homes, the result generally shows that kiln dried produced the highest heat above 21 °C, which is followed by smokeless coal (20.8 °C), seasoned wood (20.7 °C), and the wood briquette (20.6 °C) giving the least heat. This is expected because it has the least moisture content of 6%.

The average RH reduced with an increase in room volume (Fig. 2e) HO2: 39m^3^ (39 ± 4%), HO3: 57m^3^ (48 ± 2%), HO4: 74m^3^ (42 ± 8%), HO1: 86m^3^ (47 ± 3%), and HO5: 89m^3 ^(57 ± 4%) except for HO3, which could be due to its inadequate ventilation rate, while the average temperature reduces with an increase in room volume (Fig. 1e).

Regarding the stove type (Fig. 2b), Homes with open fireplaces have the highest RH (57 ± 4%), followed by eco-design (47 ± 3%), multifuel eco-design (42 ± 5%), and clear skies stage (v) stove (42 ± 2%). The mean indoor temperatures during the burning periods were higher in the home equipped with improved wood stoves: Multifuel eco-design (22.5 ± 1.6 °C), followed by clear skies stage (v) stove (21 ± 1.8 °C °C), Eco-design (20.5 ± 0.96 °C) and the least temperature in a home equipped with open fireplace (17.7 ± 2.1 °C). This finding highlighted the higher combustion efficiency of the closed stove compared to the open fireplace with lower heat dissipation. These findings are in agreement with similar studies^38^.

The woodstove’s operation kept the temperature in the room within the comfortable range, while the open fireplace did not deliver enough heat to provide thermal comfort. This highlighted the higher combustion efficiency of the closed stove compared to the open fireplace with lower heat dissipation. These findings show that the room volume, stove type, and average burning time most impacted the RH and temperature levels. Generally, the average temperature due to burning/heating was relatively higher during the burning period. Therefore, small room sizes and improved woodburning stoves are essential for a thermally comfortable room during heating periods.

### Pollutant exposure profiles in living areas

**Fig. 3:**
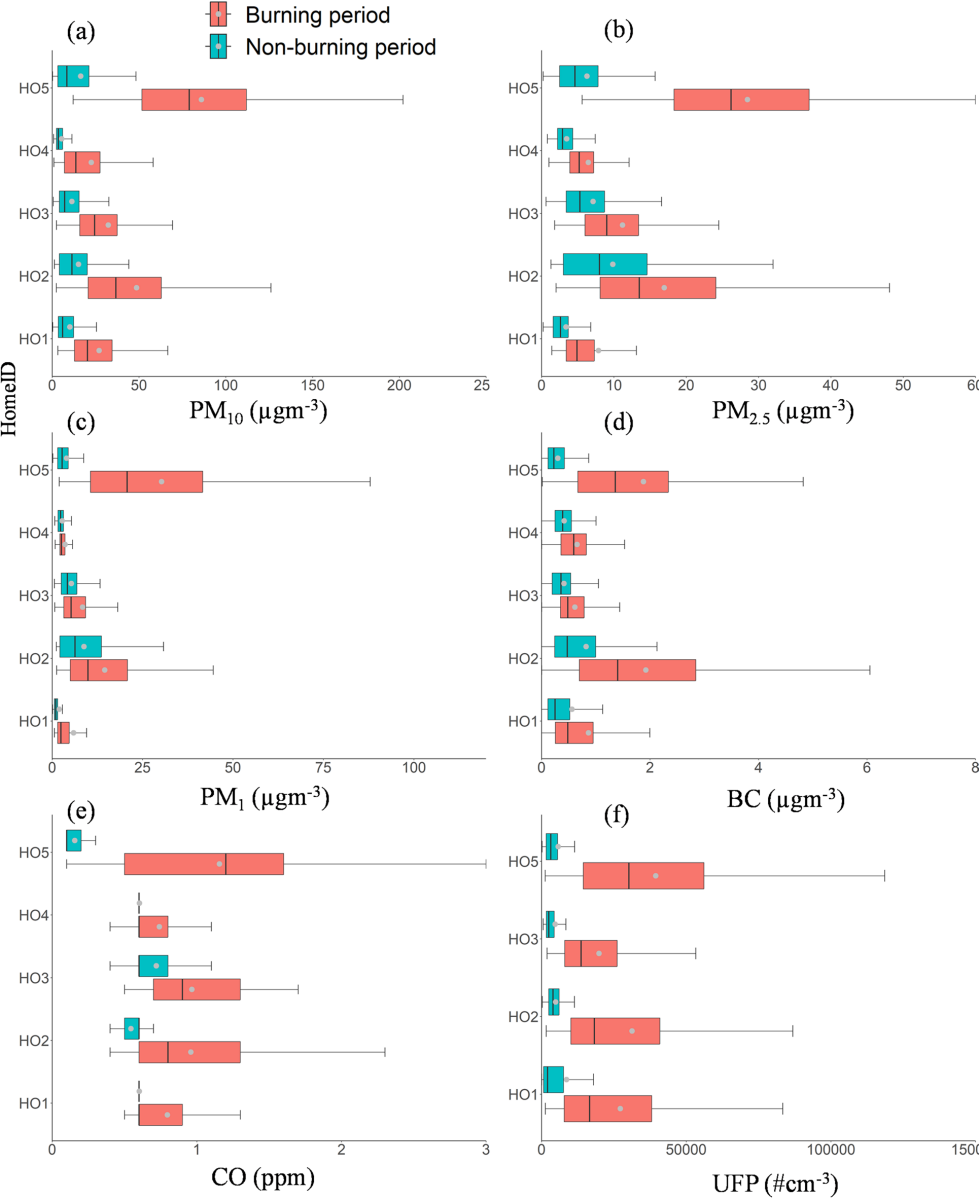
Box plots of minute average (**a**) PM_10_, (**b**) PM_2.5_, (**c**) PM_1_, (**d**) BC, (**e**) CO, and (**f**) UFP concentration during the monitoring period in each home. The plot includes the median (shown by horizontal bars), the 25th and 75th percentiles (indicated by the bottom and top edge of the boxes), and minimum and maximum values (shown by the bottom and the top edge of the whiskers).

**Fig. 4:**
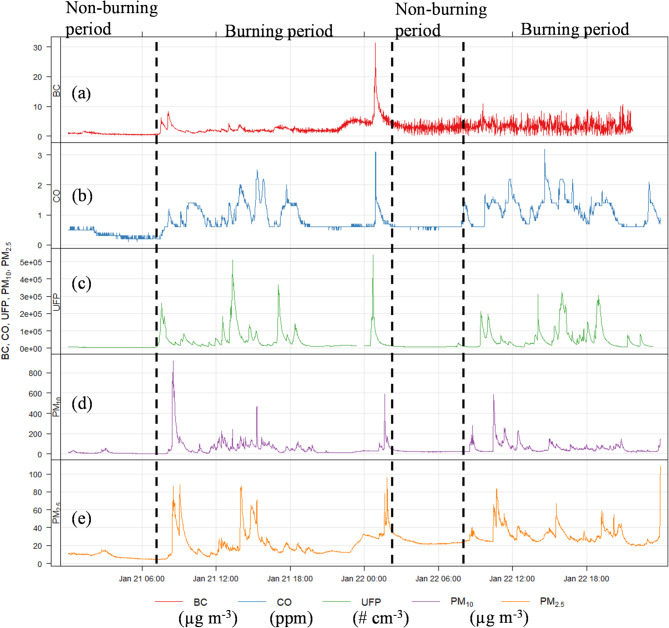
Time series showing Home2 indoor measurements of BC, CO, UFP, PM_10_, and PM_2.5_ annotated with activities that may be expected to affect pollutant concentrations. (**a**) BC, (**b**) CO, (**c**) UFP, (**d**) PM_10_, (**e**), PM_2.5_. The sampling frequency of 1 min for all pollutant concentrations was significantly higher and more variable during the burning period than in the background.

Table S4 summarises the minimum, maximum, median concentration, and interquartile range (IQR) for the whole monitoring period (burning period (BP), nonburning period (NPB), and total (24 h), for PM_10_, PM_2.5,_ PM_1,_ UFP (20–100 nm), BC, and CO in all homes. Figure 3 shows the distribution of one-minute averaged pollutant concentrations during each home’s burning and non-burning periods. Observed time series of pollutant concentrations for home users were characterised by abrupt concentration elevations within seconds of lighting the stove (Fig. 4). The peak PM_10_, PM_2.5_, PM_1_, BC, UFP, and CO concentrations were associated with lightning (ignition phase), subsequent refuelling, and residual ash removal from the wood stove, While the lowest pollutant exposure concentration was observed at midnight during non-burning periods. The t-test showed a statistical significance (*p* < 0.005) for UFP and PM_2.5_.

**Fig. 5:**
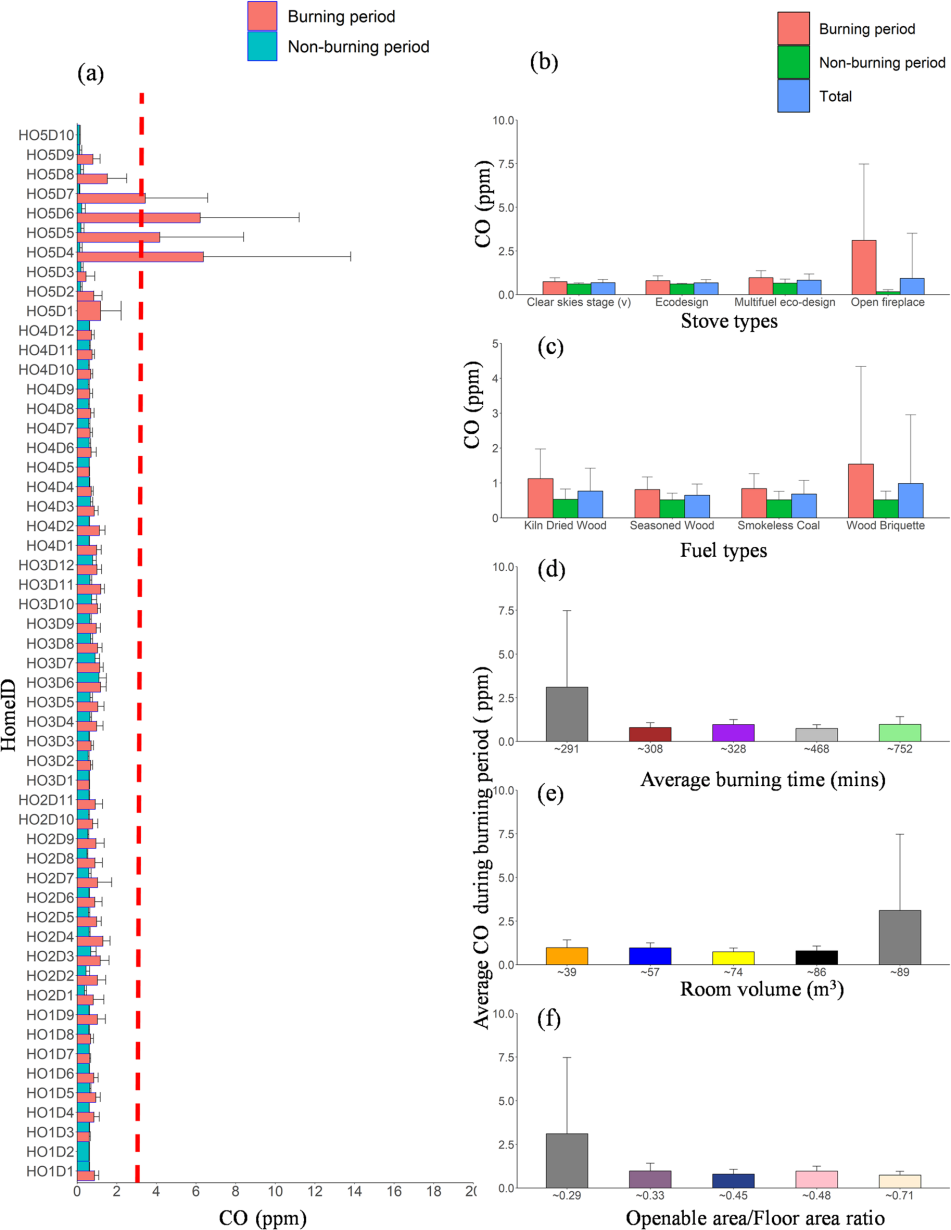
(**a**) Bar plots of CO concentrations for all homes during the burning, non-burning, and total periods. The red dotted horizontal line at ~ 3.5 ppm represents the WHO^70^ guideline for average daily CO concentration. The average CO concentration for the entire monitoring period in each home is categorised based on (**b**) stove type, (**c**) fuel type, (**d**) average burning time, (**e**) room volume, and (**f**) openable area-to-floor area ratio. Error bars indicate the standard deviation of the average values, with only positive standard deviation values shown to maintain figure clarity.

CO is a major combustion product emitted from wood burning. The background concentration of 0.6 ppm was observed during non-burning activities in HO1, HO2, HO3, and HO4, as opposed to 0.1 ppm in HO5. There was an increase in the CO concentration in the room during heating stove usage with a factor of 2 to 10, as seen by the detailed variation during the monitoring period in Figures S1-S5. This exposure concentration varied according to the stove opening (slightly or widely opened) and fire stoking to ignite solid fuel. The maximum concentration peak at 2.1, 6.7, 1.7, 1.5, and 27.6 ppm for HO1, HO2, HO3, HO4, and HO5 (Table S4; Figures S1-S5), which is below the 24 h average WHO guideline of 3.49 ppm (WHO, 2021) in the four homes with closed improved stove except for open fireplace. The highest value in HO5 could be attributed to an incomplete combustion process in an open fireplace compared with other homes with closed and improved stoves. HO4, equipped with the latest improved stove (clear skies stage (v) model), showed the lowest CO concentration, likely due to higher combustion efficiency (Fig. 5).

BC is a form of PM. It originates from incomplete combustion of carbonaceous fuel such as wood. According to the United Nations assessment of the impacts of air pollution, BC was suggested to be a better indicator of harmful PM substances emitted by combustion sources than PM_2.5_ and PM_10_^61^. The average background concentration was 0.2 µg m^−3^ between 01:00 and 7:00 h during a period without prior burning. BC concentrations during the burning periods were similar to those of urban background concentration reported in European cities (e.g., London, Barcelona, Basel^62^) with a median concentration ranging between 0.48 and 1.7 µg m^−3^. The median (maximum) BC concentrations for HO1, HO2, HO3, HO4, and HO5 were 0.5 (5.5) µgm^−3^, 1.5 (165 µg m^−3^), 0.5 (4.5 µg m^−3^), 0.6 (5.7 µg m^−3^), and 1.7 (355 µg m^−3^), respectively. This indicates that HO5, with an open fire, has the highest BC exposure, followed by HO2, which has the most extended burning period and the smallest room volume (Fig. 3).

Temporal variations of BC alongside other PM are illustrated in Fig. 4. BC closely followed the indoor particle mass concentration (PMC) and UFP patterns, with a steep decay (Fig. 4), highlighting its clear dependence and a key biomass combustion tracer. A positive correlation (*R* = 0.6) was observed between UFP and BC, indicating that PM emissions in the room were primarily from wood-burning stoves during solid fuel combustion.

**Fig. 6:**
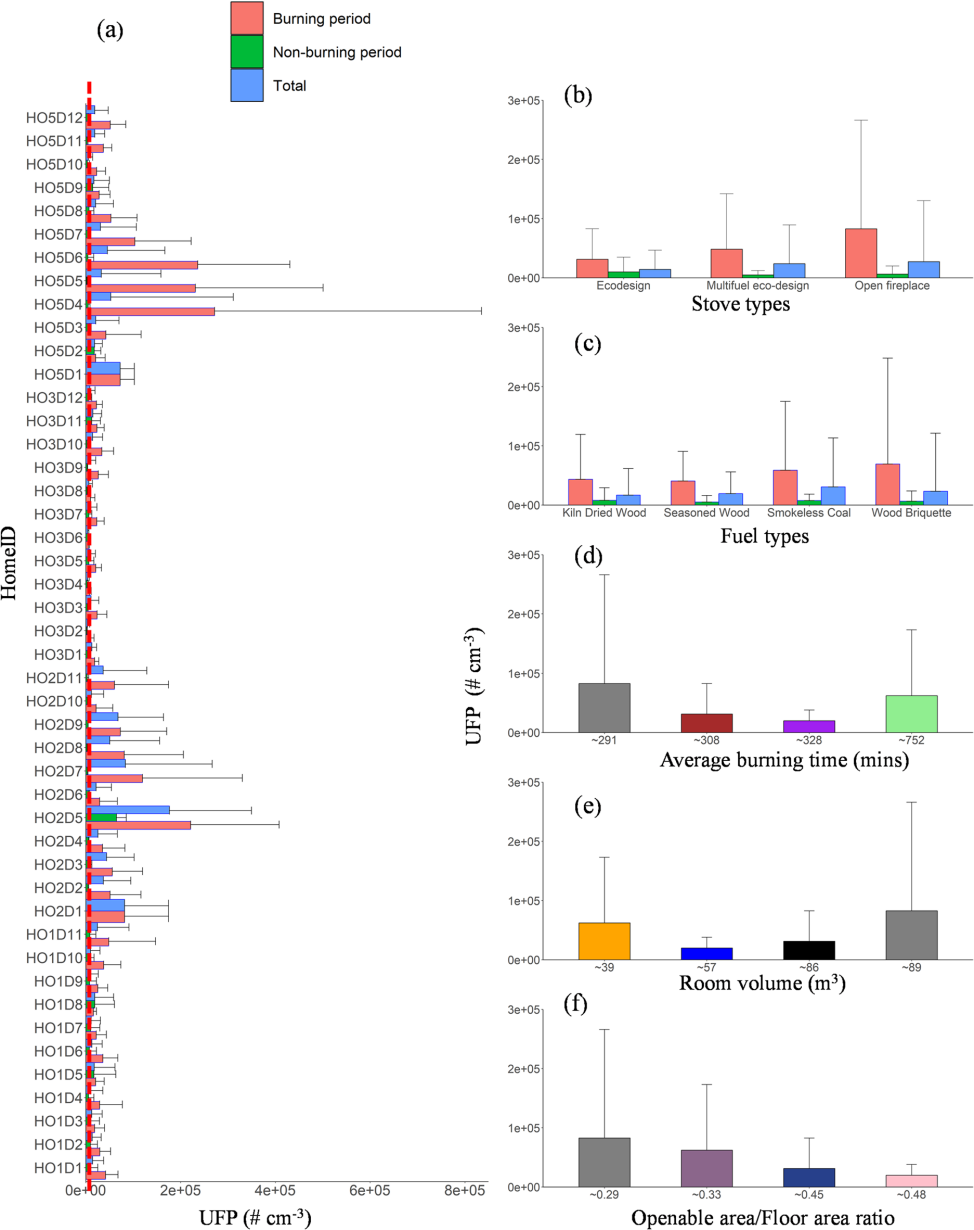
(**a**) Bar plots of UFP concentrations for all homes during the burning, non-burning, and total periods. The dashed line represents the WHO good practice statement on UFP source emission control, where > 10,000 # cm⁻³ is considered a high particle number concentration (PNC) for a 24-hour mean. The average UFP concentration for the entire monitoring period in each home is categorised based on (**b**) stove type, (**c**) fuel type, (**d**) average burning time, (**e**) room volume, and (**f**) openable area-to-floor area ratio. Error bars indicate the standard deviation of the average values, with only positive standard deviation values shown to maintain figure clarity.

A significant increase in UFP concentration was observed in all the homes during burning periods compared with non-burning periods (Fig. 6). Average UFP concentration during the burning period increased by 3.2-, 12.7-, 4.3-, and 13.3-times compared with non-burning periods in HO1, HO2, HO3, and HO5, respectively (Table S4; Figures S6-S9). Unfortunately, UFP measurements were not taken in HO4 due to instrument malfunction caused by extreme concentrations. HO4 contains a wood-burning stove in the dining area, positioned very close to the kitchen, without a vacuum extractor in use during winter. The average UFP concentration during the burning period across all homes HO1, HO2, HO3, and HO5 was 3.13 × 10^4^, 6.22 × 10^4^, 1.99 × 10^4^, and 8.26 × 10^4^ # cm^−3^, respectively (Fig. 6a). These findings align well with previous studies where similar experiments were conducted in Denmark, reporting UFP peak concentrations between 3 × 10^3^ # cm^−3^ and 2.4 × 10^5^ # cm^−3 23^; 3.9 × 10^3^ # cm^−3^ and 9.9 × 10^5^ # cm^−3 24^; and 2.6 × 10^5^ # cm^−3^ and 9.9 × 10^5^ # cm^−3 13^.

We analysed the average UFP in each home according to stove type (Fig. 6b), fuel type (Fig. 6c), room volume (Fig. 6d), and openable area/floor ratio (Fig. 6e). As for the stove type, the average UFP concentration ranged from 3.13 × 10^4^ to 8.26 × 10^4^ # cm^−3^. Average UFP concentrations were highest for open fireplaces at 8.26 × 10^4^ # cm^−3^, followed by a multifuel stove at 4.83 × 10^4^ # cm^−3^, with the eco-stove showing the lowest concentration at 3.13 × 10^4^ # cm^−3^ (Fig. 6b). This indicates that the home with open fireplaces experience higher exposure levels than those with improved, closed wood stoves. Previous studies have also demonstrated significant differences in indoor pollutant concentrations between open fireplaces and closed wood-burning stoves^38,63,64^.

As for the average burning duration in each home, the highest value of average UFPs was 8.26 × 10^4^ # cm^−3^ for HO5 (open fireplace) with the lowest average burning time (291 min day^−1^, Fig. 6d), followed by home HO2 with an improved closed stove (6.22 × 10^4^ # cm^−3^) with the highest average burning time (752 min day^−1^), and HO1 (3.13 × 10^4^ # cm^−3^; 308 min day^−1^). The lowest UFP concentration (1.99 × 10^4^ # cm^−3^) was observed in HO3 with the average burning time (328 min day^−1^). The high concentration in HO1, despite the shorter burning duration (308 min day^−1^), may be attributed to contributions from other sources, such as cooking, rather than solely from wood burning.

As for the fuel type (Fig. 6c), the average UFP concentration across all homes for seasoned wood, kiln-dried wood, smokeless coal, and wood briquette was 4.1 × 10^4^, 4.3 × 10^4^, 5.9 × 10^4^, and 6.92 × 10^4^ # cm^−3^, respectively. Wood briquettes have the highest UFP (6.92 × 10^4^ # cm^−3^), which is followed by smokeless coal, kiln-dried wood, and seasoned wood during the burning period. These results indicate that manufactured fuels (wood briquette and smokeless fuel) released higher UFP concentrations than kiln-dried and seasoned wood.

As for the room volume, the highest average UFP in HO5 at 8.26 × 10^4^ # cm^−3^ (open fireplace) despite having the largest room volume (89 m^3^ Fig. 6e). For the closed stoves, the room with the smallest room volume (39 m^3^) has the highest UFP concentration. UFP concentrations varied appreciably with different openable area/floor area ratios (Fig. 6f) because the home with the least openable area (0.29 m^2^) has the highest UFP concentration (Figure S10). In comparison, the house with the highest openable area (0.48 m^2^) has the lowest UFP concentration during the burning period.

**Fig. 7:**
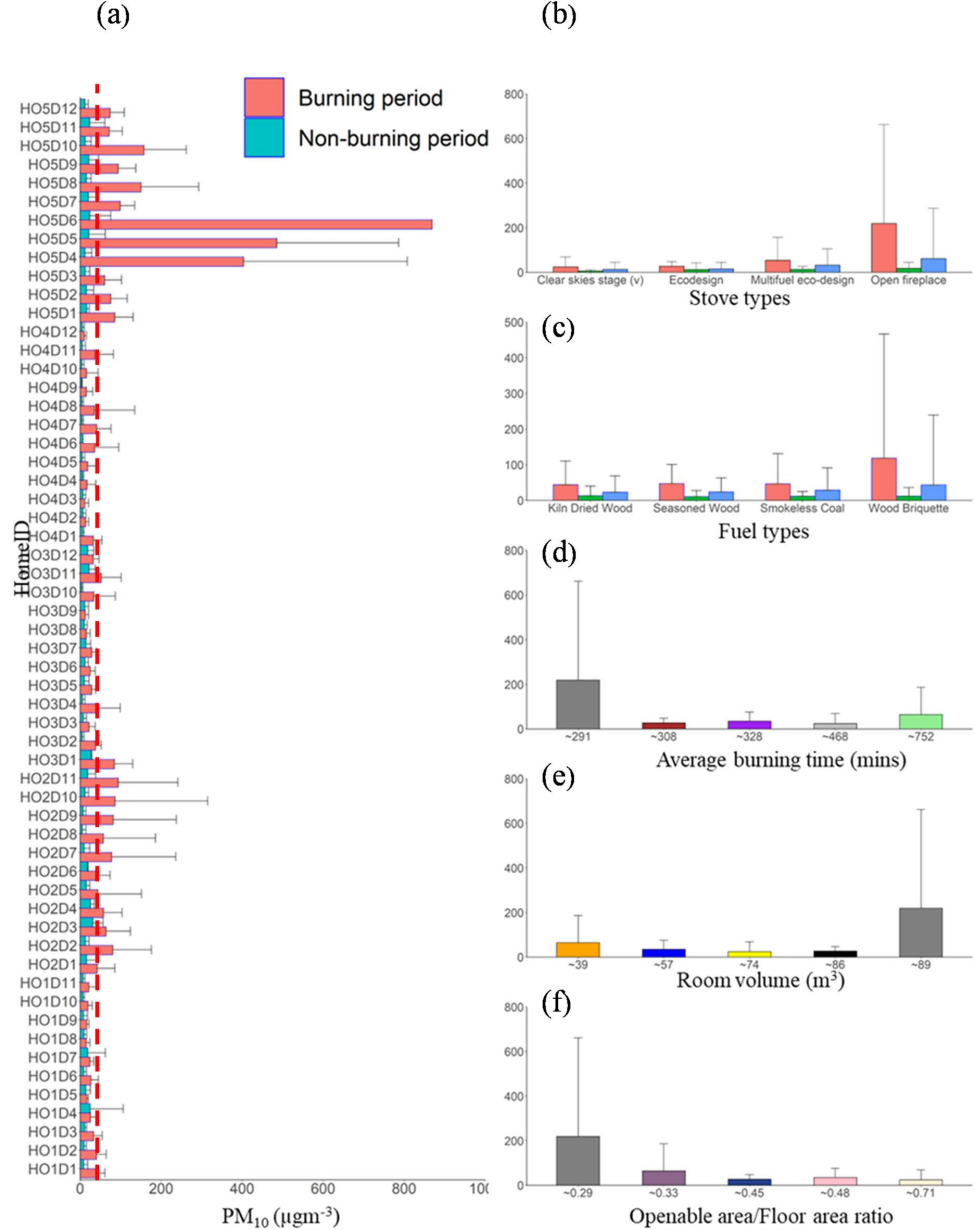
(**a**) Bar plots of PM_10_ concentration for all homes during the burning, non-burning, and total periods. The red dotted horizontal lines at 45 µg m^−3^ for PM_10_ represent the WHO^70^ guidelines for average daily PM_10_ concentration. The average PM_10_ concentration for the whole monitoring period in each home is categorised based on (**b**) stove type, (**c**) fuel type, (**d**) average burning time, (**e**) room volume, and (**f**) openable area-to-floor area ratio. Error bars indicate the standard deviation of the average values, with only positive standard deviation values shown to maintain figure clarity.

**Fig. 8:**
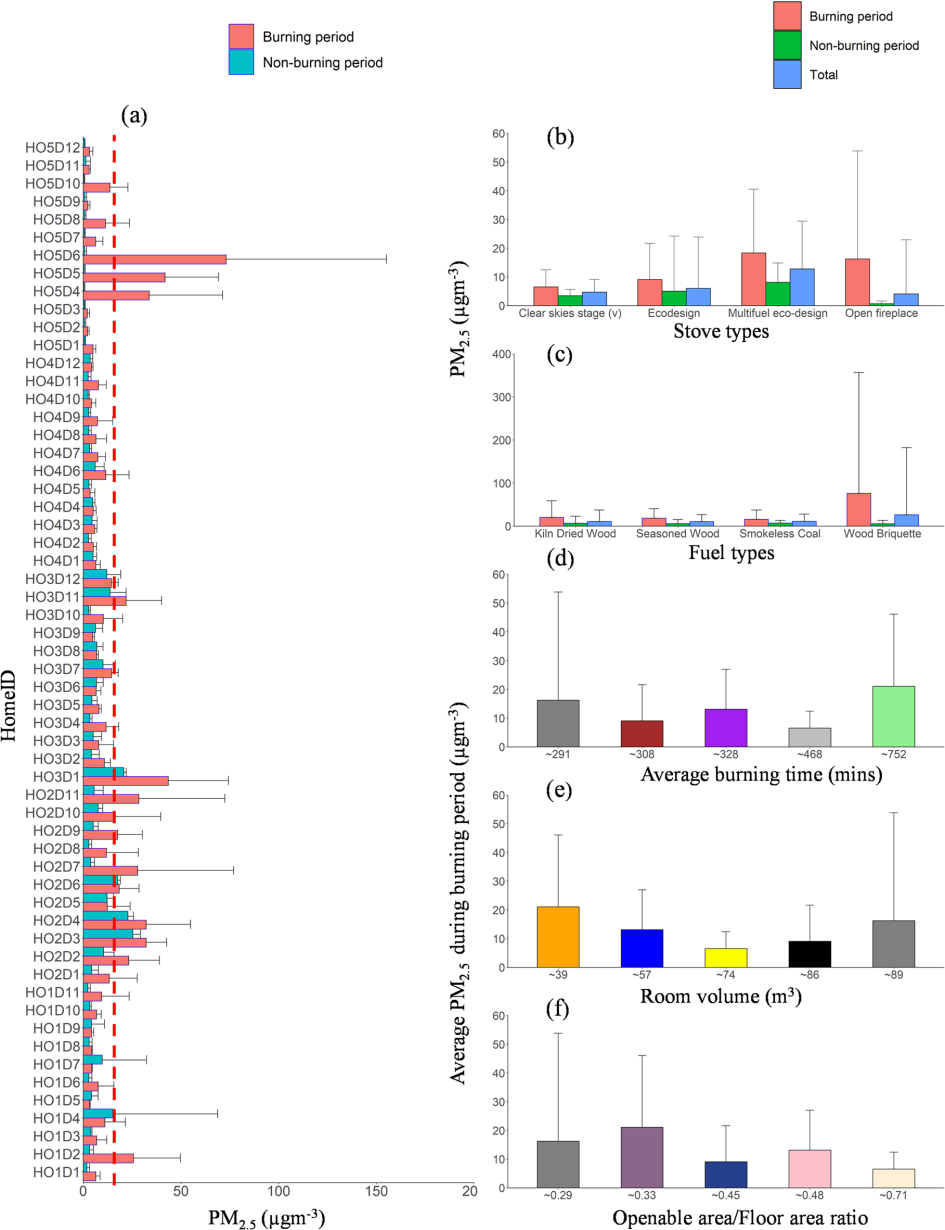
(**a**) Bar plots of PM_2.5_ for all homes during the burning and non-burning period. The blue dotted horizontal lines at 15 µgm^−3^ for PM_2.5_ represent the WHO^70^ guidelines for average daily concentration. The average PM_2.5_ concentration for the whole monitoring period in each home is categorised based on (**b**) stove type, (**c**) fuel type, (**d**) average burning time, (**e**) room volume, and (**f**) openable area-to-floor area ratio. Error bars indicate the standard deviation of the average values, with only positive standard deviation values shown to maintain figure clarity.

Regarding PMC, the PM_10_ and PM_2.5_ concentrations varied widely across homes (Table S4; Figures S11-S15) based on the factors highlighted in Sect. “Home and burning characteristics”. The median PM_2.5_ (PM_10_) concentration during the burning period varies across each home (HO1, HO2, HO3, HO4, and HO5); 4.9 (20.3) µg m^−3^, 14.2 (37.9) µg m^−3^, 9.3 (24.5) µg m^−3^, 5.2 (13.6) µg m^−3^ and 38.4 (89.6) µg m^−3^ (Fig. 4). The highest 21 (64) µg m^−3^ and lowest 6.5 (24.1) µg m^−3^. The maximum PM_2.5_ (PM_10_) concentration of 3344.8 (4117.8) µg m^−3^ and 344.5 (2962.1) µg m^−3^ was observed in HO5 and HO2 during the stove chamber cleaning of smokeless coal ash. This result is in agreement with a previous study^45^ which shows that very high concentrations of coarse and fine particles were released during residual ash removal. An assessment carried out in compliance with ISO standard 7708:1995 revealed that an individual present in a living room with an open fireplace would inhale, on average, 217 µg m^−3^ during the ignition stage and 283 µg m^−3^ during the refuelling stage^65^. The PM_2.5_ and PM_10_ levels are comparable to those of earlier studies^12,13,24,28,30,38^. Average concentration variations for each home are summarised based on the stove type, fuel type, average burning time, and room volume (Figs. 6 and 7). For comparison of the concentration of different PM sizes during total, non-burning, and burning periods, bar plots (Figs. 6a and 7a, and 8a). According to stove types, the PMC and UFP concentration followed the following order: open fireplace > multifuel stove > ecostove > clearskies stove (Figs. 6b and 7b, and 8b).

As expected, individual homes exhibited a distinct exposure concentration variation due to different stove types, burning periods, and room volume. High concentration was recorded in HO2 with a smaller room volume and highest burning duration despite having a multifuel eco-design stove. This could be due to limited dispersion, inversion buildup, and HO5 with an open fireplace. We conclude that the most important factors that influence occupants’ exposure to aerosol particles were the room volume, burning duration, stove type, fuel type, and openable area/floor area ratio. Frequent opening of both doors and windows and slight opening of the stove door during stove usage can significantly reduce the occupant’s exposure during winter.

### PM_2.5_/PM_10_ ratios

**Fig. 9:**
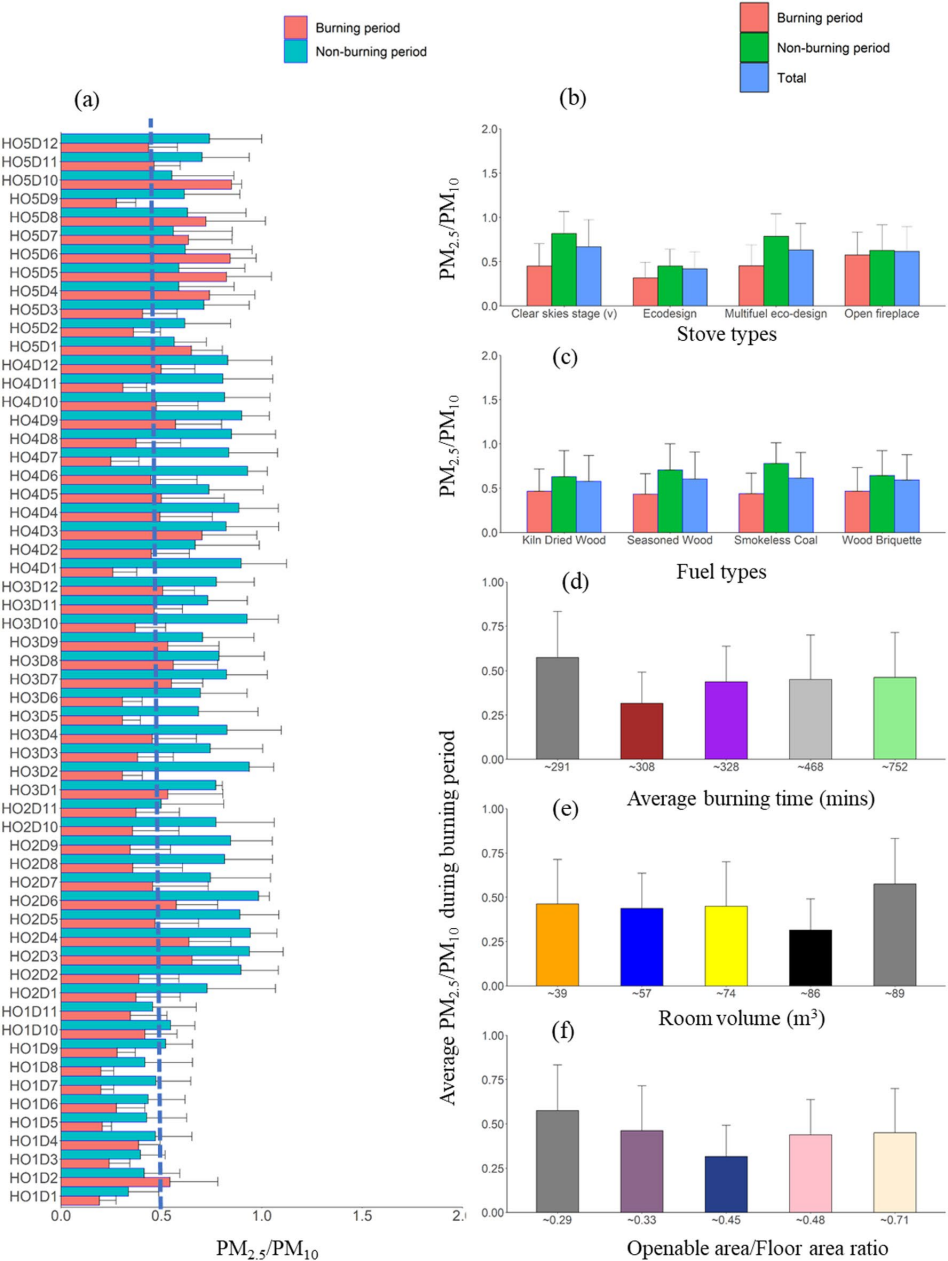
(**a**) Bar plots of PM_2.5_/PM_10_ ratios for all homes. The dashed line indicates PM_2.5_/PM_10_ of 0.5. The average ratio concentration for the whole monitoring period in each home is categorised based on (**b**) stove type, (**c**) fuel type, (**d**) average burning time, (**e**) room volume, and (**f**) openable area-to-floor area ratio. Error bars indicate the standard deviation of the average values, with only positive standard deviation values shown to maintain figure clarity.

The indoor environment is more complex and variable than the outdoor environment because there are notable variations in the sources and concentration of pollutants between and within the building. Indoor pollutant sources include building materials, consumer products, heating and cooking appliances, and other occupant activities^66^. To further understand PM pollution sources, PM_2.5_/PM_10_ ratios were estimated for each home (Fig. 9), reflecting that fine and coarse particles often originate from diverse sources beyond heating.

Average PM_2.5_/PM_10_ mass concentration ratios varied from 0.2 to 1.2, with a mean of 0.58 ± 0.04 over the study period. A significant change in this ratio was observed between burning and non-burning periods across all homes (Fig. 9a). Homes with improved closed stoves exhibited a higher ratio during the non-burning period, except HO1, indicating a greater contribution of PM_2.5_ to PM_10_, likely from activities such as cooking or vacuuming. This suggests that improved stoves reduce PM_2.5_ concentration during burning. To find the association between the different sizes of the PM, linear regression analysis showed a significant correlation between PM_2.5_ and PM_10_ (*r* = 0.69; *p* < 0.001), as expected since PM_2.5_ contributes more to PM_10_ throughout the monitoring period. As shown in Fig. 9, fine particles (PM_2.5_/PM_10_ > 0.5) were dominant in all homes during non-burning periods, except for HO1, while PM_2.5_/PM_10_ < 0.5 was evident on 90% of the monitoring days during burning periods. HO5 was an exception, as it has an open fireplace and shows PM_2.5_/PM_10_ > 0.5 on 80% of the monitoring days.

The average PM_2.5_/PM_10_ ratio was analysed by stove type (Fig. 9b), fuel type (Fig. 9c), room volume (Fig. 9d), and openable area/floor ratio (Fig. 9e). For stove type, the PM_2.5_/PM_10_ ratios ranged from 0.32 ± 0.25 for eco-stoves to 0.45 ± 0.25 for multifuel and 0.57 ± 0.26 for open fireplaces showing the highest emissions of PM_2.5_.

Regarding fuel type (Fig. 9c), smokeless coal had the highest PM_2.5_/PM_10_ ratio (0.62 ± 0.29), followed by seasoned wood (0.61 ± 0.3), wood briquettes (0.59 ± 0.28), and kiln-dried wood (0.58 ± 0.29), indicating that smokeless coal produces a more significant proportion of PM_2.5_.

The average PM_2.5_/PM_10_ in each home was also analysed in relation to average burning time. HO5, with the least burning duration (291 min day^−1^), had the highest PM_2.5_/PM_10_ ratio (0.57 ± 0.26). In contrast, HO2, with the longest average burning time (752 min day^−1^), had a lower ratio of 0.46 ± 0.25 (Fig. 9d). The ratios for HO4 (468 min day^−1^) and HO3 (328 min day^−1^) were 0.45 ± 0.25 HO4 and 0.44 ± 0.2, respectively, HO1 had the lowest ratio (0.32 ± 0.18) despite an average burning time of 308 min day^−1^. Since the burning durations were generally comparable, a clear trend regarding the effect of burning duration on the PM_2.5_/PM_10_ ratio was not apparent.

When analysed by room volume, HO2, with the smallest room (39 m^3^), has the highest PM_2.5_/PM_10_ ratio of 0.46 ± 0.25 (Fig. 9e), while HO1, with the second largest room (86 m^3^), had the lowest ratio (0.32). In contrast, HO5 has the highest ratio (0.57 ± 0.26) despite having the largest volume (89 m^3^). This aligns with findings from similar studies on kitchen^66^ where larger volume kitchens exhibited higher PM_2.5_/PM_10_ ratios.

PM_2.5_/PM_10_ ratios did not significantly vary with different openable area/floor area ratios (Fig. 9f). For all the homes studied (HO1, HO2, HO3, HO4, and HO4), the openable area/floor area ratios (PM_2.5_/PM_10_ ratios) were 0.45 (0.32), 0.33 (0.46), 0.48 (0.44), 0.7 (0.45), and 0.29 (0.57). HO5, with the least openable area/floor area ratio (0.29), had the highest PM2.5/PM10 ratio (0.57), while HO4, with the openable area/floor area ratio, had a PM_2.5_/PM_10_ ratio of (0.45) during burning period. Thus, the primary factors influencing the PM_2.5_/PM_10_ ratio are stove type, fuel type, and room volume.

### Room characteristics and CO_2_ concentrations

**Fig. 10:**
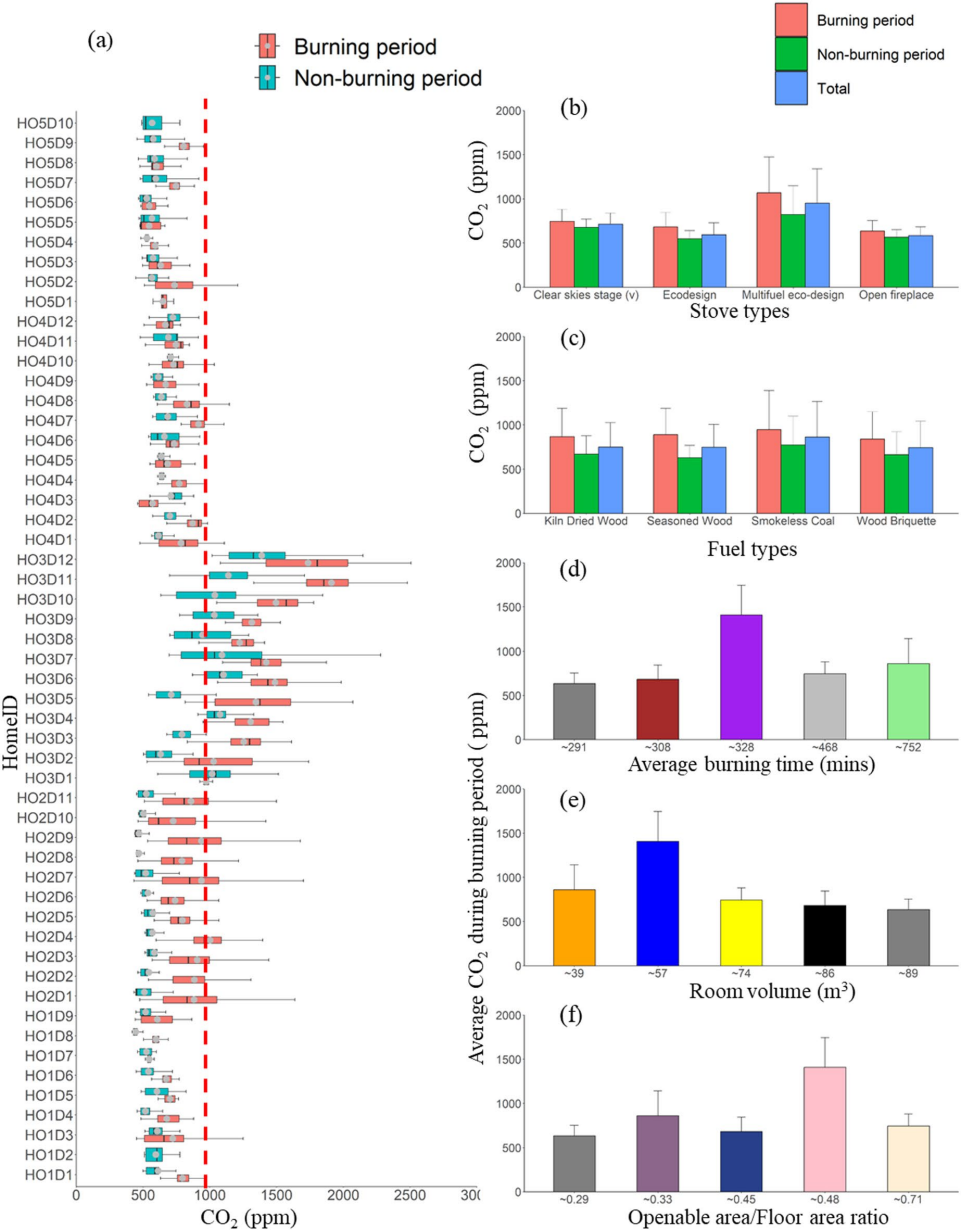
(**a**) Boxplot of the daily CO_2_ concentrations for each of the investigated homes. The dashed red line at 1000 ppm indicates the corresponding reference CO_2_ concentration ^*100*^. The average CO_2_ concentration for the whole monitoring period in each home is categorised based on (**b**) stove type, (**c**) fuel type, (**d**) average burning time, (**e**) room volume, and (**f**) openable area-to-floor area ratio. Error bars indicate the standard deviation of the average values, with only positive standard deviation values shown to maintain figure clarity.

CO_2_ is a reliable indicator of ventilation and is influenced by factors such as occupancy, occupancy duration, room volume, ventilation rate, and combustion activity. Across all homes, the average room CO_2_ concentration during the non-burning periods (Fig. 10a) was 662 ± 130 ppm, rising by 30.6% to 865 ± 208 ppm during burning periods. Most homes showed average CO_2_ concentrations between 634 and 860 ppm, except HO3, which reached 1407 ppm during burning, likely due to higher occupancy and low ventilation. Besides ventilation, occupancy, room volume, and fuel types also impact CO_2_ concentrations.

For fuel type effects, the average CO_2_ concentration during burning (912 ± 347 ppm) was ~ 92 ppm higher than the overall mean concentration of 816 ± 320 ppm (Fig. 10b). All homes used four fuel types except HO1, which used three due to the stove type. HO2 recorded higher CO_2_ concentrations due to longer burning times. Grouped by fuel type, smokeless coal showed the highest CO_2_ concentration (982 ± 456ppm) as opposed to the kiln-dried wood, showing the lowest (887 ± 330 ppm) during burning. HO1, which did not use smokeless coal, had the lowest CO_2_ concentrations, unlike homes with multifuel and clear skies Stage (v) stoves that used all four fuel types. CO_2_ emissions are closely linked to combustion efficiency, fixed carbon content, and oxygen availability^67^. Energy-efficient stoves, such as Clear Skies and multi-fuel models, enhance combustion, reducing CO_2_ emissions. Improved stoves burn fuel more effectively, lowering CO_2_ emissions.

Figure 10c groups homes by room volume (m^3^) as follows: HO1 (86m^3^), HO2 (39m^3^), HO3 (57), HO4 (74), HO5 (88.5), with average CO_2_ concentrations during burning of 665 ± 90 ppm, 861 ± 258 ppm, 1375 ± 212 ppm, 748 ± 101 ppm, and 634 ± 120 ppm, respectively. Overall, CO_2_ averages were lower in larger rooms with more openable areas (e.g., HO5 at 634 ± 120 ppm, HO1 at 681 ± 164 ppm, and HO4 at 744 ± 137 ppm), indicating an association between room volume and lower CO_2_ concentrations. This aligns with in-kitchen studies^66^ where large-volume kitchens (46-120m^3^) had 20–28% lower CO_2_ concentrations than smaller ones. Therefore, new home designs should prioritise placing wood stoves in rooms with greater volume and openable areas.

Regarding the burning time, HO1, with the shortest burning duration (291 min day^−1^), had the lowest CO_2_ concentration during burning (634 ± 120ppm), as opposed to HO2, with the longest burning duration (752 min day^−1^). Still, the least occupancy showed an average CO_2_ concentration of 860 ± 282 ppm. HO3 had the highest CO_2_ concentration during burning due to high occupancy.

Occupancy significantly impacts CO_2_ concentrations (Fig. 10d). Homes with more than two occupants during burning sessions have 50% higher average CO_2_ concentrations than those with one occupant, demonstrating that CO_2_ concentrations rise with occupancy^68^. These results suggest limiting active occupancy in rooms with wood burners to minimise CO_2_ build-up. Variations in CO_2_ concentrations followed occupancy patterns, peaking between 07:00 and 10:00 h and 15:00 and 20:00 h due to occupant respiration and limited ventilation (doors/windows closed in winter).

The average background CO_2_ concentration was 493 ppm (measured between 00:00 and 07:00 h), while the hourly average reached 884 ppm, exceeding SAGE’s recommended 800 ppm (SAGE, 2021). Elevated CO_2_ concentrations largely reflect occupant respiration and limited ventilation, as a single occupant exhales ~ 20 L of CO_2_ per hour, equivalent to 180 ppm CO_2_ per hour. Outdoor CO_2_, not measured here, is typically assumed to be ~ 400 ppm in urban settings.

### ACH and VR

**Fig. 11:**
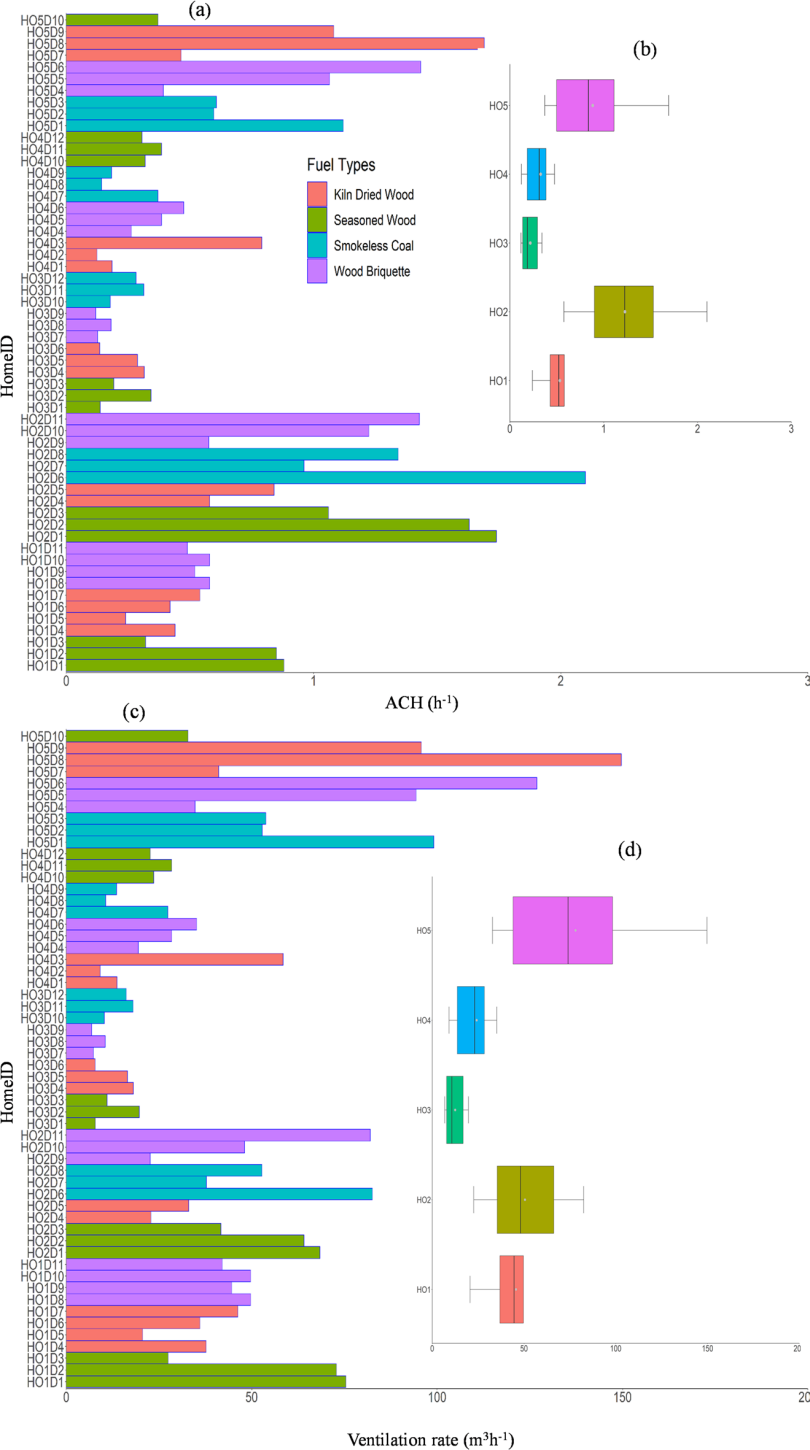
(**a**) Bar plots of the daily ACH (h^−1^) in all homes based on the fuel types, (**b**) box plot of a minute average across each home, (**c**) bar plots of the daily VR (m^3^ h^−1^) for all homes based on fuel type, and (**d**) boxplot of one minute average VR (m^3^ h^−1^) across all homes.

All the homes are naturally ventilated with air change rates controlled by opening windows and doors without heating, ventilation, and air conditioning (HVAC) systems in the studied rooms. These spaces do not have exhaust fans like kitchens but feature chimneys for stoves, providing a draft. Figure 11 presents the average ACH values in five ranges (low, bare minimum, good, excellent, and ideal) calculated using the approach outlined in Sect. “Room characteristics and CO_2_ concentrations”^69^. All the homes have low ACH (< 3 h^−1^). This was expected as doors and windows remain closed in winter to conserve energy, limiting ventilation. HO3 showed the ACH, indicating an airtight space that could increase occupant exposure.

The average ACH (VR) in HO1, HO2, HO3, HO4, and HO5 are 0.53 h^−1^ (46 m^3^h^−1^), 1.23 h^−1^ (50.5 m^3^h^−1^), 0.22 h^−1^ (12.5 m^3^h^−1^), 0.33 h^−1^ (24 m^3^h^−1^), 0.79 h^−1^ (69 m^3^h^−1^), respectively. These ACH values are similar to those obtained in German (0.21–0.27 h^−1^) and Spanish (0.718–0.779 h^−1^) homes with woodburning stoves during winter^24,38^. HO2 has the highest ACH, likely due to its small room volume (39 m^3^), followed by HO5 and HO1. Despite closed doors and windows, these old homes still allow air exchange, but HO3 and HO4, fitted with modern sealed doors and windows, show significantly lower ACH (< 0.5 h^−1^). Room volume and ACH greatly influence pollutant concentration, aligning with studies linking lower ACH and VR values to higher PM levels. Increased VR and ACH can help reduce PM and CO_2_, creating a healthier indoor environment. Grouped by volume, HO2 with the smallest room (< 40 m^3^) had the highest average ACH (1.67 ± 0.76 h^−1^), followed by HO4.

### UFP hazard ratio

**Fig. 12:**
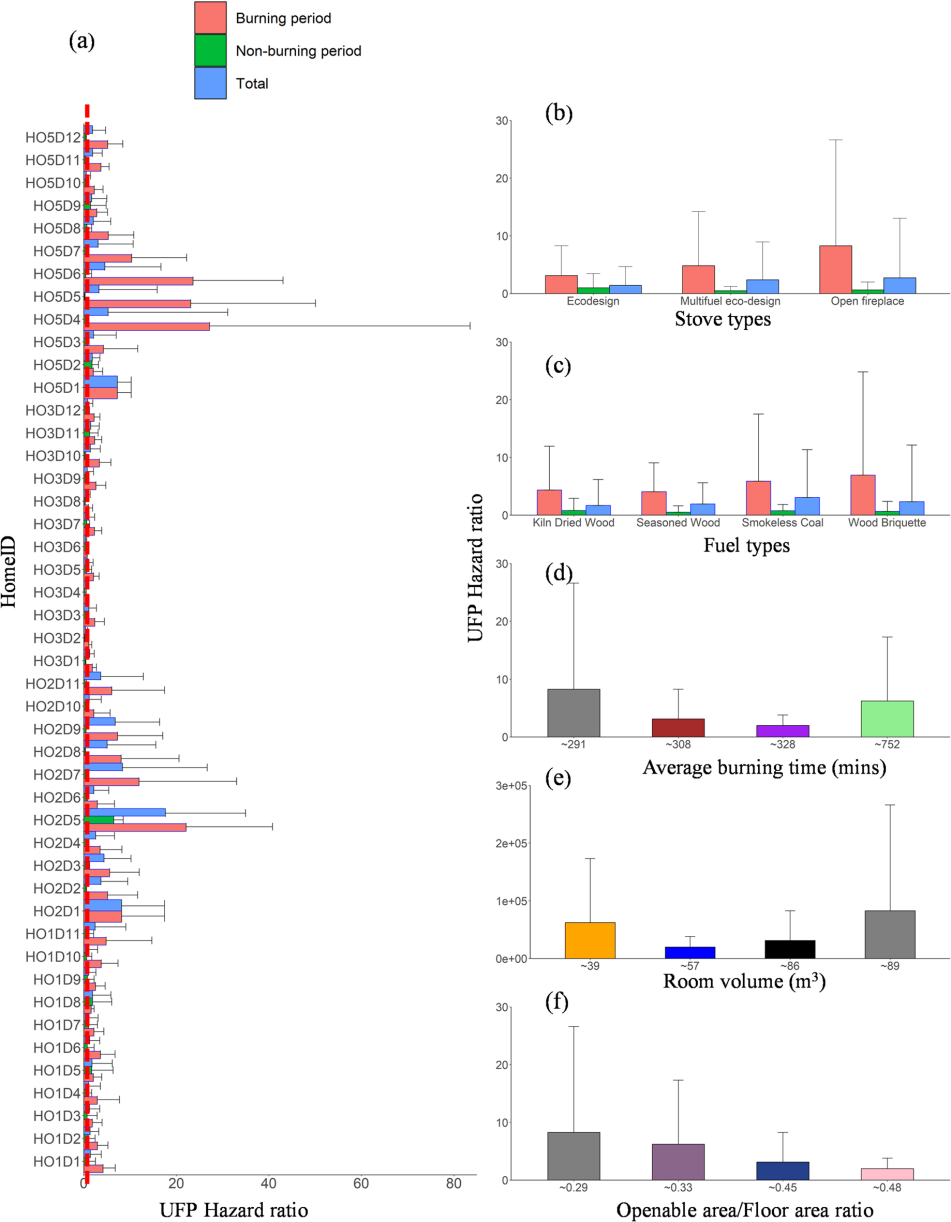
(**a**) Bar plots of UFP hazard ratios for all homes. The dashed line indicates a UFP hazard ratio of 1. The average ratio for the whole monitoring period in each home is categorised based on (**b**) stove type, (**c**) fuel type, (**d**) average burning time, (**e**) room volume, and (**f**) openable area-to-floor area ratio. Error bars indicate the standard deviation of the average values, with only positive standard deviation values shown to maintain figure clarity.

UFP remains an unregulated pollutant due to the absence of national or international guidelines. However, the WHO has issued a good practice statement on UFP to guide national and regional authorities and research toward measures to reduce ambient UFP: concentrations < 100 # cm^−3^ are considered low, while > 10,000 # cm^−3^ (24-hour mean) or 20,000 # cm^−3^ (1-hour mean) are regarded as high^70^. Our HR estimates were based on 24-hour mean UFP concentration values exceeding 10,000 # cm^−3^, with HR varying across home environments. Figure 12 shows the HR bar plot by home, stove type, room volume, fuel type, average burning duration, and openable-to-floor area ratio. The highest HR was observed in HO5 (8.3), followed by HO2 (6.2) and HO1 (3.1), with the lowest HR in HO3 (1.9). By stove type, open fireplace showed an HR of 2.8, followed by 2.4 and 1.4 for the multifuel eco stove and an eco-design stove, respectively. Smaller room volumes (< 40 m³) had the highest HR (6.2) due to limited space for dispersion. Among fuel types, wood briquettes had the highest HR (6.9), followed by smokeless coal (5.9), kiln-dried wood (4.3), and seasoned wood (4.1). Although all fuels tested are considered “clean”, none achieved an HR of 1 for the 24 h average.

## Summary and conclusion

We conducted a comparative assessment of occupant exposure in Guildford homes using improved wood-burning stoves and open fireplaces with clean solid fuels (seasoned wood, kiln-dried wood, smokeless coal, and wood briquette). The study examined the role of room volume, stove type, fuel type, burning duration, and ventilation rate on indoor air quality and thermal comfort. The key conclusions drawn are as follows:

- PM_10_ exposure during burning increased twelve-fold for open fireplaces and three-fold for improved stoves compared to non-burning periods. Pollutant concentrations were highest during fire lighting, refuelling, stoking, and ash cleaning. Cooking and vacuuming also contributed to IAQ degradation during non-burning periods. Homes with improved stoves-maintained temperatures (21.3 ± 1.5 °C) within the thermal comfort range, while open fireplaces resulted in lower temperatures (17.7 ± 2.2 °C). Relative humidity varied between 44.3 ± 2.93% and 57.3 ± 4.2% for improved stoves and open fireplaces, respectively.
- All studied homes had low ACH, below 3 h^−1^, likely due to closed doors and windows in winter for energy conservation, resulting in limited ventilation and lower ACH.
- Higher UFP exposure was linked to longer burning durations, smaller room volumes (< 40m^3^), and frequent chamber openings. Open fireplaces resulted in the highest UFP exposure, followed by multifuel eco-design stoves.
- Manufactured fuels, such as smokeless coal and wood briquettes, emitted UFP concentrations at twice the level of wood fuels like kiln-dried and seasoned wood. Additionally, PM_2.5_ exposure was 3.7 to 4.1 times higher when burning wood briquettes compared to kiln-dried or seasoned wood, challenging claims that manufactured fuels are cleaner alternatives. This may be due to their difficulty in ignition, often requiring multiple natural firelighters, which significantly contribute to indoor pollutant emissions.
- HO1 and HO3 exhibited lower HR due to shorter burning periods (as wood burners were secondary heating sources). HO2 and HO5 had higher HR values for UFP, with all homes exceeding the WHO good practice statement for UFP during burning periods.
- Regarding heat released, kiln-dried wood produced the highest heat across all homes and with the least pollutant emission. This may be due to its lower moisture content. Burning clean, solid wood can pose health risks for occupants, so short-term usage is advised if it must be utilised.
- During winter, ventilation is limited, and most open areas are shut to conserve energy. The most effective way to reduce personal exposure during heating is by using sustainable methods, such as solar-powered or electric heaters. However, many people are transitioning to single-space heating with wood-burning stoves due to rising energy prices and inflation.

Based on our findings, we recommend the following to reduce occupant exposure during wood stove use.

- **Periodic window opening reduced the PM**_**2.5**_ **and PM**_**10**_ **exposure by about 2.9- and 1.8-times**,** respectively**,** compared to homes with closed windows**. HO1, which practised window opening during wood burning and cooking, regardless of stove and fuel type, exhibited lower pollutant concentrations. Natural ventilation can significantly improve IAQ but relies on clean outdoor air. Behavioural interventions, such as regularly opening windows, especially during non-occupancy periods, are advisable during potentially higher emission events, such as stove cleaning, lightning, and refuelling. Additionally, using an ash vacuum cleaner during stove cleaning should be encouraged to reduce PM_10_ and PM_2.5_ exposure levels further.
- **Homes with small living room volumes (< 39 m**^**3**^**) showed higher pollutant exposure levels**,** even when using improved eco-design stoves and cleaner solid fuels**,** compared to those with larger volume rooms**. The limited volume in smaller rooms restricts pollutant dispersion. Existing homes can benefit from increased natural or mechanical ventilation and the use of air purifiers during wood burning to mitigate exposure. Increasing ceiling height, room size, and window size for new home designs can enhance pollutant dispersion and improve IAQ.
- **Using seasoned wood and kiln-dried wood significantly reduced average PM**_**2.5**_ **exposure during burning compared with manufactured fuels (wood briquettes)**. PM_2.5_ exposure concentrations for occupants during wood briquettes were 4.1- and 3.7 times higher than for seasoned and kiln-dried wood. Although manufactured fuels are often marketed as cleaner alternatives, they do not emit lower pollutants than traditional seasoned and kiln-dried wood.
- **The use of open fireplaces significantly increased median PM**_**10**_, **PM**_**2.5**,_ **exposure during the burning periods – by 3.7-and 6.4-times – for homes with closed improved eco-design and 4.1-and 7.4-times for Clear Skies Stage (v) stoves respectively.** Occupants using open fireplaces experienced the highest pollutant exposure concentrations. In contrast, improved stoves with additional secondary and tertiary air supply facilitated better fuel combustion, reducing PM exposure concentration in indoor spaces.
- **Longer burning durations (all-day burning) should be avoided when using wood-burning stoves**, **as extended use results in the highest particle exposure.** Homes with multifuel eco-design stoves as the primary heat source exhibited high UFP exposure concentrations comparable to those in homes with open fireplaces as secondary heat sources. Low-cost, energy-efficient air purifiers can be used to improve IAQ, particularly since ventilation is often limited.

While this study addressed occupant exposure in homes using clean solid fuels and eco-design stoves for space heating compared to conventional open stoves, filling essential gaps regarding UFP hazard ratios and potential inhaled doses, it found that exposure levels during wood stove use vary based on stove type, fuel type, room volume, and burning duration. Further studies should explore air purifier effectiveness on various air pollutants, including UFP concentrations and their associated chemical composition from wood-burning sources to better estimate health impacts.

## Methodology

### Study design

**Fig. 13:**
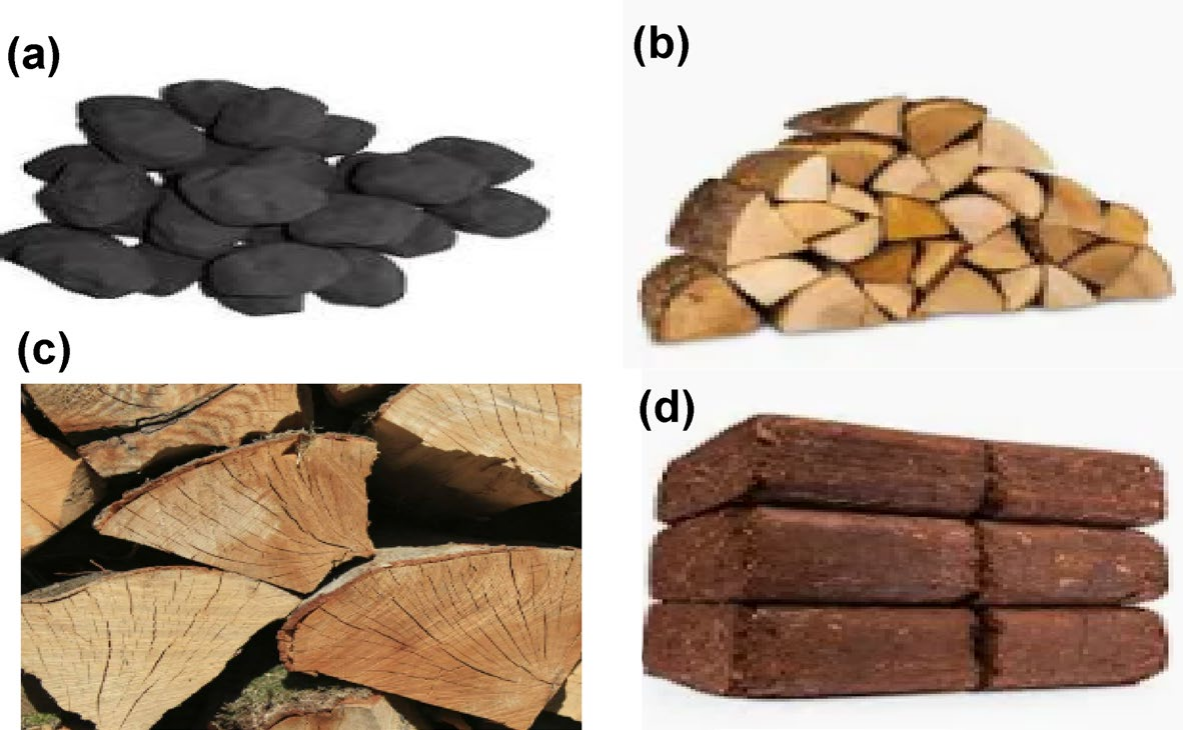
Images of different solid fuel types used in the field experiments, including (**a**) smokeless coal, (**b**) kiln-dried wood, (**c**) seasoned wood, and (**d**) wood briquette.

This study was designed to assess indoor air pollution exposure from wood-burning stoves using various fuel types in real-world conditions during winter in a typical UK town, Guildford^71^. The consideration was given for replicating the same experiment in 5 homes (see Figure S16). In each house, twelve days of continuous monitoring were carried out in the living area where the burning stove was installed. We considered five types of burning stoves and four types of fuels (Fig. 13), as listed in Tables 1 and 2. The monitoring included the following variables: UFP (20–100 nm), PMC (PM_2.5_ and PM_10_), BC, CO, and CO_2_ (see Sect. “Room characteristics and CO_2_ concentrations”). The qualitative data were also collected via designing building and occupant surveys to provide detailed structural and occupants’ burning activities. Table S2 shows an overview of building surveys, which considered building type, building location, floor area, room volume, window size, door dimensions, monitoring location, and ventilation conditions. The occupant survey covered factors such as the number of room occupants during burning, type of fuel used and wood burning stove, time and duration of burning, and status of natural ventilation during the burning period.

**Table 1:** Details of the studied homes, showing characteristics such as the sampling duration, fuel type, burning duration, stove location, and stove and ventilation types. All the house has natural ventilation. Note: KD: kiln dried fuel; sw = seasoned wood; sc = smokeless coal; wb = wood briquette.

Home ID	Room volume L (m)×W(m)×H(m): (m^3^)	ACH (h^−1^)	Fuel type	Sampling period	Stove type	Mean burning duration per day (mins)	Age of stove (year)	Stove location	Chimney sweep (months)	Ventilation type	Primary heating sources	Secondary heating sources
HO1	7.6 × 4.9 × 2.3 (85.7)	0.52	KD, SW, SC	24.12.2022–14.01.2023	Eco-design stove	308	5	Living room	6	Natural	Oil	Wood
HO2	3.6 × 4.2 × 2.6 (39.3)	1.23	KD, SW, SC, WB	19.01.2023–30.01.2023	Multifuel eco-design	752	0.1	Dining room	12	Natural	Wood	Gas
HO3	6.2 × 3.7 × 2.5 (57.4)	0.21	KD, SW, SC, WB	01.02.2023–14.02.2023	Multifuel eco-design	328	6	Living room	6	Natural	Electric	Wood
HO4	7.6 × 2.7 × 3.6 (73.9)	0.33	KD, SW, SC, WB	19.02.2023-02.03.2023	Clear skies stage (v)	468	1	Dining/kitchen room	6	Natural	Electric	Wood
HO5	5.2 × 6.3 × 2.7 (88.5)	0.88	KD, SW, SC, WB	09.02.2024–21.02.2024	Open fireplace	291	> 50	Living room	6	Natural	Wood	Electric

**Table 2:** A fuel-stove combination used during indoor air quality monitoring in each home.

House number	Stove type	Fuel type	Fuel moisture content (%)	Operation	Nominal heat output (kW)	Days of monitoring
HO1	DEFRA-approved (Eco design stove)	Locally sourced seasoned wood	13	Closed	5	3
		Wood briquette	5.8	Closed	5	3
		Kiln-dried wood	8.1	Closed	5	3
HO2	DEFRA-approved (Multifuel eco stove)	Locally sourced seasoned wood	13	Closed	5	3
		Smokeless coal	12.6	Closed	5	3
		Wood briquette	5.8	Closed	5	3
		Kiln-dried wood	8.1	Closed	5	3
HO3	DEFRA-approved (Multifuel eco stove)	Locally sourced seasoned wood	13	Closed	5	3
		Smokeless coal	12.6	Closed	5	3
		Wood briquette	5.8	Closed	5	3
		Kiln-dried wood	8.1	Closed	5	3
HO4	DEFRA-approved (Clear skies stage (v) stoves)	Locally sourced seasoned wood	13	Closed	5	3
		Smokeless coal	12.6	Closed	5	3
		Wood briquette	5.8	Closed	5	3
		Kiln-dried wood	8.1	Closed	5	3
HO5	Open fireplace	Locally sourced seasoned wood	13	Open	5	3
		Smokeless coal	12.6	Open	5	3
		Wood briquette	5.8	Open	5	3
		Kiln-dried wood	8.1	Open	5	3

To ensure comparability of results across all studied homes, we confirmed that the following specified criteria were met in each home (Sect. “Study area”): (i) non-smoking homes (i.e., with no smokers); (ii) they were either on the ground or first floor; (iii) had a living room with an operational wood heating stove equipped with different types of heating stoves (open fire and improved stoves such as eco-design stoves and clear skies stage (v) stoves); (iv) each home should have a minimum of one or more occupants; (v) homeowners burned the provided solid fuel (KDW, SW, SC, and WB) in their typical burning routine, with wood burning occurring daily during the monitoring period; (vi) there was space for monitors, which were placed at breathing height (1.5 m above ground level) and 1.5 m away from the wood burner, using the same measuring instruments to eliminate equipment bias; and (vii) the monitoring duration for each home was consistent. Before starting the study, ethics approval was obtained from the University of Surrey’s Research Ethics Board.

### Study area

Since 1 January 2022, the UK Department for Environment, Food, and Rural Affairs (DEFRA) has enforced the Ecodesign Directive (EU 2015/1185) for solid fuel stoves, introducing stricter efficiency and emission standards. Requirements include a minimum efficiency of 75%, maximum particulate matter emissions of 40 mg/m³, carbon monoxide emissions of 0.12%, and organic gaseous carbon limited to 120 mg/m³. DEFRA-approved stoves, also known as “smoke control exempt appliances”, should meet these standards and can be used legally in “Smoke Control Areas”. These stoves are optimised for burning authorised fuels, such as dry, seasoned wood (moisture < 20%) and smokeless fuels, ensuring reduced particulate matter and gaseous emissions^72,73^. Guildford’s study area is situated in Southeast England’s Surrey County. It is also listed as the UK’s ‘smoke control area,’ meaning that only authorised fuel and DEFRA-approved appliances are allowed^74^.

#### Characterisation of the study area

The study area, Guildford, United Kingdom, is a mid-sized town in southeastern England. As of 2024, Guildford has a population of approximately 682,235^75^. The town’s climate is classified as C_f_b (temperate, fully humid, warm summer) under the Köppen climate classification system. It is characterised by mild winters and moderate precipitation throughout the year^76^ and an annual mean temperature of 10.6 °C, with average high temperatures of 22.8 °C in summer and average lows near 1.7 °C in winter. The town receives an average annual precipitation of about 663 mm^76^.

The primary emission sources in Guildford include transportation, residential heating, and industrial activities^77^. Notably, the use of wood-burning stoves for domestic heating has been increasing, likely due to rising energy costs and a growing preference for wood stoves as a supplementary heat source.

#### Biomass use in industrial processes

In the UK, biomass is used as a renewable energy source in various industrial processes, including power generation, manufacturing, and agriculture^78^. Within the study area, biomass use is predominantly limited to residential wood burning rather than large-scale industrial applications. However, nearby industrial facilities may contribute additional emissions depending on their reliance on fossil fuels or biomass as part of their energy mix. In Guildford, biomass is used for boilers and heating purposes, although the statistics are unknown. A comprehensive understanding of biomass usage and emissions in the study area is critical for assessing the impacts of local air quality and informing policy recommendations.

#### Description of studied homes

Five homes with wood-burning stoves were monitored (Table 2). They were chosen based on specific criteria (Sect. “Study design”). The stoves were installed on the ground floors, and four fuel types (KD, SW, SC, WB) were used, except in HO1, which had an eco-design stove incompatible with SC fuel. While a summary is given below, detailed characteristics of each home and plan can be found in Table S5.

- Home 1 (HO1): Located in East Shalford Village, Guildford, this 4-bedroom detached house has oil-based central heating and an eco-design wood stove used as secondary heating in the evening. The living room (7.6 m×4.9 m×2.3 m) has three windows and a wooden floor with a central carpet.
- Home 2 (HO2): A 3-bedroom semi-detached house in Normandy, Guildford, near a moderate-traffic road. It uses a multifuel eco-design stove as the primary heating source on the ground floor. The dining area (4.29 m×3.62 m×2.5 m) has wooden floors, one window, and a door leading to other rooms. The stove operates daily from morning until midnight.
- Home 3 (HO3): A 4-bedroom detached house in Merrow, Guildford, near a major road. It has electric central heating and a small multifuel eco-design stove used as secondary heating in the living room (6.2 m×3.7 m×2.5 m). The stove operates mainly in the evenings.
- Home 4 (HO4): A 3-bedroom detached house in Onslow, Guildford, near the A3 heavy-traffic road. The home uses a DEFRA-approved Clear Skies stove (2022 model) in a combined dining/kitchen room (7.6 m×2.7 m×3.6 m). Due to high ultrafine particle (UFP) concentrations, UFP data could not be collected.
- Home 5 (HO5): Located in Bramley Village, this 400-year-old, 5-bedroom historical house has an open fireplace used as secondary heating in the evenings. The living room (5.2 m×6.3 m×2.7 m) has a wooden floor, three windows, and two doors.

### Instrumentation

The experimental setup included two optical particle spectrometers (Testo-350 DISCmini, GRIMM model 11-C), Aethalometer AE51, and TSI Q-Trak monitors) to measure indoor UFP, PM_10_, PM_2.5_, PM_1_, BC, CO, CO_2_, temperature, and relative humidity at 1-min logging intervals. The same GRIMM 11 C and GRIMM 11D (GRIMM Aerosol Technik GmbH & Co KG, Ainring, Germany) were used to monitor each home. Detailed information on the sampling frequency, flow rates, manufacturer, model, measurement range, and uncertainties for all instruments has been presented in Table 3. to increase readability. All instruments were factory-calibrated before the field campaign and had been used in prior studies^79^. Before deployment at each home, all the equipment was routinely cleaned and filters changed. The Met Office obtained hourly averaged meteorological parameters (wind speed, wind direction, and rainfall) for weather stations closest to the monitored home.

**Table 3:** The detailed information on the sampling frequency, flow rates, manufacturer, model, measurement range, and uncertainties for all instruments used during the indoor air quality monitoring in each home.

Instrument type	Sampling frequency	Flow rates	Manufacturing and model measuring range	Uncertainties	Wavelength	Variables
Aethalometer AE51	1 min	100 mLmin^−1^	AETHLABS; microAeth^®^ AE51; 0–1 mg m^−3^	± 0.1 µg m^−3^	880 nm	BC
Testo-350 DISCmini	1 min	1.0 L min^−1^ ±0.1 L min^−1^	TESTO; 20 nm: 2E3 −1E6 # cm^−3;^ 100 nm: 5E2-5E5 # cm^−3^	± 30% in size and number of typical	-	UFP
GRIMM model 11-C/11D	1 min	1.2 L min^−1^	GRIMM Aerosol Technik; 11 C &11D; 0.22–32 μm; 0.1–100,000 µg m^−3^	± 3%	660 nm	PM_10_, PM_2.5_, PM_1_
TSI Q-Trak	1 min	-	TSI Q-trak; Model 7575; Probe models 982; 0–500 ppm	± 3% of reading or 3 ppm	-	CO
	1 min	-	0–5000 ppm;	± 3% or ± 50 ppm	-	CO_2_
	1 min	=	0–60 ^o^C	± 0.5^o^C	-	Temperature
	1 min	=	5–95% RH	± 3% RH (includes ± 1% hysteresis	-	RH

### Data collection

The field campaign took place continuously in each home between December 2022 and February 2024 (for 24 h in 12 days) during the winter season. One-minute logging intervals of UFP, PM_2.5_, PM_10_ and BC were collected for 12 days in the 5 Guildford homes (Tables S1 and S2), giving up to 60 days of total monitoring across all homes. Measuring instruments were placed at the average adult breathing height of 1.5 m above the floor and approximately 1.5 m from the wood-burning stove. The monitors were resets between residences. Each volunteer signed an informed consent form and completed a building and occupancy survey form. In addition, homeowners kept records of burning activities and other home activities during the monitoring periods. The record offered helpful information that improved comprehension of pollution sources and exposure circumstances.

The field researchers collected qualitative information about the building, occupants, and the outdoor surrounding area (Table S2) during the monitoring period. The building surveys provided an overview of factors, such as building location, home configurations, window and door dimensions, apartment type, floor number, number of rooms, ventilation conditions, floor area, room volume, window size, door dimensions, and monitoring location. The occupant survey covered factors such as the number of occupants, type of heating stoves, fuel used, and time and duration of burning; the survey also shows other sources of combustion besides woodburning, including cleaning and cooking. Room temperature and humidity data were also simultaneously recorded (Table S3), alongside the information on outdoor sources of pollution (such as traffic, industrial sites, idling vehicles, and outdoor domestic burning) and their proximity to the studied homes. Data was regularly downloaded from the instrument throughout the monitoring period for compilation.

### Determination of air change rates (ACH) and ventilation rates

ACH is the number of times the air is entirely replaced per hour in an indoor environment and is a key parameter affecting indoor microenvironments’ air quality and energy consumption. To estimate personal exposure to different pollutants, it is crucial to know the ACH value, which is calculated using CO_2_-based methods, and three different methods, steady state, decay, and build-up, are used to establish the occupancy phase or concentration trend^80–82^. Also, the level of ventilation is indicated by the ACH value^83,84^.

The steady-state and build-up approaches apply when at least one person uses the living area, while the decay method is used in an unoccupied home^80,81^. The ACH values were classified into five categories: low (< 3 h^−1^), bare minimum (3–4 h^−1^), good (4–5 h^−1^), excellent (5–6 h^−1^), and ideal (> 6 h^−1^). The decay and build-up methods were applied to the CO_2_ time series during occupancy periods. The build-up method was chosen because combustion activities greatly affected the CO_2_ concentrations, and the decay method was used because occupancy is not required. In contrast, the steady-state method was not adopted because it was impossible to maintain CO_2_ levels while people were burning in the living area for extended periods.

Decay ACH (A_D_) is estimated when CO_2_ decays from its peak concentration and stabilises at ambient concentrations. AD (h^−1^) was calculated using Eq. (1):

1 $$\:{A}_{D}=\frac{1}{\varDelta\:t}\text{ln}\left\{\frac{{C}_{1}-{C}_{R}}{{C}_{0}-{C}_{R}}\right\}T$$

Where ∆t = time (h), between two observations, the difference in CO_2_ concentration between C0 and C1 (ppm; values recorded at the beginning and end of the observation time, respectively), and CR is the steady state concentration at the lower occupancy (ppm), or the lowest CO_2_ concentration in the air replacement In contrast to other methods, the decay method is not constrained by variables like the number of inhabitants, room volume. The following criteria were followed for each decay sequence: A maximum of 8 h of data are required to ensure representative, reliable, and efficient data collection while minimising errors from long-term variations or measurement noise; C1 must achieve a steady state and be close to background CO_2_ concentration, while ∆CO_2_ must be more significant than 100 ppm to ensures that the collected data remains accurate and exceeds the instrument’s resolution limit^80^.

Build-up ACH (AB): AB (h^−1^) was estimated using the build-series of CO_2_ concentrations and Eq. (2):

2 $$\:{A}_{B}=1/\varDelta\:t\:\:\text{l}\text{n}\left\{\right({C}_{SS}-{C}_{0})/({C}_{SS}-{C}_{1}\left)\right\}$$

Where ∆t = time (h) between C_0_ and C_1_, i.e., the initial and the final CO_2_ concentration (ppm) recorded during the observation. Equation (3) was used to calculate the final steady-state concentration at equilibrium state CSS (ppm).

3 $$\:{C}_{SS}=({{C}_{b}}^{2}-{C}_{0}{C}_{1})/(2{C}_{b}-{C}_{0}-{C}_{1})$$

Where C_b_ is the CO_2_ concentration (ppm) at the midpoint of the observation period, the build-up method is only applicable to time series if the following conditions are met: (i) C_0_ should be near the CO_2_ concentration in replacement air; (ii) CSS cannot be negative; and (iii) the method only functions for data collected in 20 min, because the 20-minute restriction optimises the method by focusing on the period where CO₂ concentration rises meaningfully, avoids potential inaccuracies due to steady-state or noise, and maintains consistency with assumptions about mixing and external conditions^80,81,85^. Additionally, it was ensured that for both methods, the correlations (R^2^) between the measured and projected concentrations were ≥ 0.9.

Adequate ventilation removes stale indoor air in exchange for clean outdoor air^86,87^. Therefore, it is important to estimate the impact of ventilation rate on IAQ, the occupant’s well-being, energy use, and heat loss. Ventilation rates (Q) in m^3^h^−1^ were estimated using Eq. (5)^88^:

4 $${\text Q}={\text A}{\text C}{\text H}\times{\text V}.$$

ACH (h^−1^) is the air exchange rate calculated in Sect. “Determination of air change rates (ACH) and ventilation rates”, and *V* is the room volume (m^3^).

### Hazard ratio assessment

The hazard ratio (HR) of each pollutant was estimated by dividing the mean concentration by its corresponding reference concentration (RfC)^89^ using Eq. (5).

5 $$\:{HR}_{i}={C}_{i}/Rf{C}_{i}$$

Where $\:{C}_{i}$ = measured 24 h average concentration of a pollutant and RfCi = corresponding reference concentration. Reference values of 15 and 45 µgm^−3^ were used for PM_2.5_ and PM_10_ for the 24 h averages, and 10,000 #cm^−3^ for UFP^90^.

### Data analysis

The collected data were pre-processed to identify and remove instrument errors, missing data, and reading errors through logbook reviews, outliers’ detection, and temporal anomalies. The cleaned data were averaged every 1 h. Data analysis was then performed on the data set using QGIS, Microsoft Excel, and statistical computing software R^91^ with the help of the open-source package Openair^92^. All statistical significance tests were 2-tailed, and the confidence index was 95%. A level of p-value ≤ 0.05 was considered to be statistically significant. Descriptive statistics were shown as median with interquartile range.

## Electronic supplementary material

Below is the link to the electronic supplementary material.

Supplementary Material 1

## Data Availability

Data is provided within the manuscript or supplementary information files in the form of tables and figures.
