# Supplementary material for: Particulate matter exposure from different heating stoves and fuels in UK homes
Source: Sci Rep. 2025 Jul 1;15:21394. doi: 10.1038/s41598-025-05886-1 (PMC12217894; doi:10.1038/s41598-025-05886-1)
Supplement: Supplementary file 1 — Supplementary Material 1 [file 41598_2025_5886_MOESM1_ESM.docx]

**Supplementary Information**

for

**Particulate matter exposure from different heating stoves and fuels in UK homes**

Abidemi Kuye^a^, Prashant Kumar^a,b,*^

^a^Global Centre for Clean Air Research (GCARE), School of Engineering, Civil and Environmental Engineering, Faculty of Engineering and Physical Sciences, University of Surrey, Guildford GU2 7XH, United Kingdom

^b^Institute for Sustainability, University of Surrey, Guildford GU2 7XH, Surrey, United Kingdom

*Corresponding author. Address as above. E-mail addresses: P.Kumar@surrey.ac.uk, Prashant.Kumar@cantab.net

**Table S1.** Summary of previous relevant field studies on indoor air pollution in homes with residential heating stoves.

| Country  (Publication Year) | Pollutants monitored | Focus area and key findings | References |
| --- | --- | --- | --- |
| Portugal  (2023) | PM_2.5_ | - Assess the emission rate and factors affecting PM_2.5_ emission from fireplaces and woodstoves. - Woodstoves and fireplaces are estimated to be 21.6mg/kg and 58.0mg of PM_2.5_ per kg of firewood. | Martins, et al. ^1^ |
| Portugal  (2022) | PM_2.5,_ VOC and CO | - Evaluated the influence of different heating systems on indoor air quality in 25 homes. - Highest pollutant concentrations were observed in homes with open fireplaces; maximum hourly concentrations for PM_2.5_ and CO were 1,500 µg m^-3^ and 80 ppm, respectively. - VOC concentrations mainly were associated with cleaning products, air fresheners, and burning incense. | Feliciano, et al. ^2^ |
| USA  (2022) | PM_2.5_ and Levoglucosan | - Tested the correlation between indoor air pollution and woodburning stove age by measuring PM_2.5_ and levoglucosan concentrations in 30 homes. - Maximum PM_2.5_ concentration ranged from 100 to >3000 µg m^-3^. - Levoglucosan concentrations showed a linear correlation (*R*^2^ =0.92) with the total PM_2.5_ collected on the filters. - Neither mean peak PM_2.5_ nor levoglucosan concentrations were correlated with the stove age (*R*^2^ ≤ 0.07, *p* > 0.23). - PM_2.5_ and Levoglucosan concentrations were correlated with flue cleaning rather than wood stove age. | Rahman, et al. ^3^ |
| USA  (2021) | PM_2.5_ | - Studied the relationship between indoor PM_2.5_ concentrations and the household and stove-use characteristics. - Homes whose chimneys have not been cleaned in a year had 65% higher PM_2.5_ concentration than those cleaned within 6 months. - Households with low and medium-quality stoves had 161% and 186% higher PM_2.5_ concentrations than homes with higher-quality stoves (95%). - 39 % of the 1-minute PM_2.5_ observations exceeded the USEPA annual health-based standard for ambient air. - Indoor PM_2.5_ concentrations were above 12 µg m^-3^ which is the USEPA health-based annual ambient air pollution. - Indoor air quality can be improved with regular chimney cleaning and good stove quality. | Walker, et al. ^4^ |
| UK  (2020) | PM_2.5_ and PM_1_ | - Monitored indoor PM concentration in homes with DEFRA-approved stoves. - Maximum peak average of 47.60 µg m^-3^ and 36.15 µgm^-3^ for PM_2.5_ and PM_1_ and some users could be exposed to maximum PM_2.5_ concentrations of 160 µgm^-3^. - Daily average indoor PM concentration was 196.23% and 227.8% higher for PM_2.5_ and PM_1,_ respectively, when a stove was used, compared with homes without wood-burning stoves. - Hourly peak averages of PM_2.5_ and PM_1_ were 123.9% and 133.09% higher than daily averages_._ - Peak from the hourly average concentrations depended on the number of fuel pieces burned at a time and the length of the burning period. - Occupants with domestic stoves were at risk of exposure to high concentrations of PM_2.5_ and PM_1_ in a short period during normal usage. | Chakraborty, et al. ^5^ |
| USA  (2020) | PM_2.5,_ BC, OC, EC | - Examined the associations of wood stove use with PM_2.5_ mass and BC in 137 pregnant women's homes. - Median (IQR) for PM_2.5_ and BC concentration was 6.65(5.02)µgm^-3^ and 0.23(0.20) µgm^-3^ across all homes. - PM_2.5_ and BC concentrations were 20.6% and 61.5% higher in homes with stoves compared to homes without. - Homes with wet or mixed wood, non-EPA-certified stoves, and older stoves were correlated with higher pollutant concentrations, especially BC. | Fleisch, et al. ^6^ |
| Portugal  (2020) | CO, CO_2_, and PM_10_ | - Monitored the indoor air quality in two uninhabited homes with open fireplaces and wood stoves under closed doors and windows. - Average indoor temperature was lower in a room with a fireplace (14±1.6°C) compared to a room with a woodstove (20±0.94°C) - A sudden increase in CO concentration was observed for both appliances with a peak concentration of 3.20ppm on an 8-h average for an open fireplace. - Peak PM_10_ concentration was observed during the ignition and refuelling stage for both stove types, with a higher peak observed at the ignition phase. - Estimated PM_10_ emission rates from the open fireplace were higher (1.29 ± 1.25 mg min^−1^), compared with that of woodstove operation (0.049 ± 0.035 mg min^−1^). | Vicente, et al. ^7^ |
| Norway  (2019) | Fine PM, UFP, CO_2_ | - Monitored the air quality for 3h, including the background concentration, the light-up, burning, and refill phases. - Peak of fine and UFP emissions are connected to the opening of the wood stove door i.e., during light-up and refilling. - Compared to peak acceptable PM concentrations, ultrafine PM concentrations were higher and occurred frequently due to other sources and took a longer time to return to background concentration | Hamon, et al. ^8^ |
| Italy  (2018) | PM_10,_ PM_2.5_ | - Assessed indoor quality of two flats with closed improved heating stove - Collected PM samples from indoor and outdoor areas of each flat - Biomass fuel heating systems are a significant source of indoor pollution mainly due to stove cleaning and ash removal (particles in the fine and coarse size range) - Average concentration of Cu and Mn in PM_10_ and PM_2.5_ during the study period was six times more than (Cu), and about twice (Mn) concentrations measured outdoors | Frasca, et al*.*^9^ |
| Italy  (2016) | PM_10,_ PM_2.5,_ UFP, PAHs, TVOC | - Monitored discontinuous and continuous IAQ in homes with fireplaces and woodburning stoves - Minimum, maximum, and average values of the 12-h PM_10_ concentration were 16.8, 350.7 and 68.6 µgm^-3^, respectively. - Average benzo[a]pyrene 12h concentration was 9.4 ngm^-3^, while the maximum and the minimum values were 24.0 ngm^-3^ and1.5 ngm^-3^, respectively. | De Gennaro, et al. ^10^ |
| Norway  (2016) | PM_2.5_ | - Monitored indoor PM_2.5_ concentration in 36 households with wood stoves and other combustion sources for 7 days. - Average hourly PM_2.5_ concentration was higher in 14 homes with wood stoves (15.6 µgm^-3^) than in 22 homes without wood stoves (12.6 µgm^-3^). | Wyss, et al. ^11^ |
| USA  (2015) | PM_2.5_, PNC | - PM_2.5_ was monitored indoors and outdoors in USA rural homes with residential wood heating systems. - Average PM_2.5_ concentration exceeded the WHO air quality guidelines. - Household income was inversely proportional to PM_2.5_ and PNC concentrations. | Semmens, et al. ^12^ |
| Portugal  (2014) | PM_2.5_ | - Monitored indoor PM_2.5_ concentration in a primary school with a wood heating stove. - Source apportionment showed that wood burning and resuspension of soil and chalk contribute to 60% of the total PM_2.5_ in the classrooms. | Canha, et al. ^13^ |
| Germany  (2014) | PM_2.5_, CO, PAH, Formaldehyde (HCHO), acetaldehyde (CH_3_CHO), Benzene, NOx, CO_2_, TVOC, UFP | - Studied the effect of using wood wood-burning fireplaces on indoor air quality. - Wood-burning fireplaces were found to be a significant source of UFP. - Increased CO and NOx emission was observed for oven one during the ignition phase due to an atmospheric inversion restricting the air draught from the combustion chamber into the chimney. - Oven 3 had the peak concentration in most pollutants monitored during the firing period: NO_2_, HCHO, CH_3_CHO, Benzene, PM_2.5_, and UFPs are 0.016mgm^-3^, 0.055ppm, 89µgm^-3^, 72 µgm^-3^, 55 µgm^-3^, and 988354 #cm^-3^, respectively. | Salthammer, et al. ^14^ |
| Denmark  (2013) | UFPs | - Evaluated the effect of wood-burning stoves on thermal comfort and particle pollution in the indoor environment. - Findings show that opening the stove door during reloading was the primary source of UFP emissions. - Peak concentrations were 7-90 times higher than the house's background UPF concentrations; they ranged from 0.24-2.16×10^11^ # m^-3^. - After the stoves were lit, air change rates rose between 2% and 90%. | Carvalho, et al. ^15^ |
| Canada  (2009) | PM_2.5_ | - Monitored PM_2.5_ concentration in the outdoor and indoor air at 15 homes for 6 days before and after stove exchange. - Average indoor PM_2.5_ concentration during the pre-exchange and post-exchange sampling period was 12.8µgm^-3^ and 12.2µgm^-3^, respectively. - No consistent relationship was found between stove technology and indoor air quality improvements. - Indoor sources of PM_2.5_ contributed majorly (65% of the total indoor PM_2.5_ concentration), irrespective of a stove technology upgrade. | Allen, et al. ^16^ |
| USA  (2008) | PM_2.5_, organic/elemental carbon (OC/EC) | - Assessed the changes in IAQ when old stoves were replaced with USEPA-certified wood stoves. - PM_2.5_ was monitored in 16 homes before and after stove replacement. - Results showed a 71% and 76% reduction in average and maximum PM_2.5_ concentration, respectively. - 45% reduction in Levoglucosan with EPA-certified wood stoves. | Ward, et al. ^17^ |
| Ireland  (2008) | CO, PM_2.5_, PM_10_ | - Studied the impact of fireplaces on the indoor air quality of 26 Irish homes. - Estimated particle emission rates for mass concentration using the mass balance equation were 0.20 mgmin^-1^ and 0.66 mg min^-1^  for PM_2.5_ and PM_10_, respectively. | Guo, et al. ^18^ |
| Sweden  (2008) | PAHs | - Indoor and outdoor concentrations of 27 PAHs were measured in homes with and without wood-burning stoves. - Outdoor concentrations of all PAHs except for the methylated phenanthrenes were higher than indoor levels. - Indoor concentrations of PAHs in 13 homes with woodburning stoves were 3-5 times higher than in homes without woodburning stoves. | Gustafson, et al. ^19^ |
| Denmark  (2006) | PM_2.5_, CO, NO, NO_X_ | - Investigated the contribution of residential wood combustion to ambient air quality areas with high wood combustion appliance users. - Higher PM_2.5_ and EC concentrations were found with a diurnal pattern in the evening and morning peak, which are predicted times for the start of wood combustion. - Average hourly difference between PM_2.5_ concentration measured in the background and the residential area with wood combustion was 4.4 µgm^-3^. | Glasius, et al. ^20^ |

Table S2. The qualitative information of the homes and occupant survey template.

|  | Indoor Monitoring | | | | | | | | |
| --- | --- | --- | --- | --- | --- | --- | --- | --- | --- |
|  | City-Home ID code:  Home location/area (Lat, Long):  Floor #:  Home type:  Stove location:  Building material of house:  Number of people in household: | | | | | | | | |
|  | Week #:  Starting – End date: | | | | | | | | |
|  | Room type and size (Length x Width x Height):  Location of window (height from floor and ceiling):  Size of window:  Location of door/window/monitor:  Size of door:  Floor type:  Note: please provide high-level schematic of kitchen and the photos – location of stove relevant to window, door and monitor | | | | | | | | |
|  | Monitor location (height from floor): | | | | | | | | |
|  | Type of stove (or type of fuel):  Stove size/capacity:  Existence of ventilation devices:  Were ventilation devices on during monitoring:  Internal heating/cooling devices at home:  Were Internal heating/cooling devices at home on during monitoring: | | | | | | | | |
| Data | |  | Day 1 | Day 2 | Day 3 | Day 4 | Day 5 | Day 6 | Day 7 |
| Heating duration (hh:mm) | |  |  |  |  |  |  |  |  |
| Total time spent heating (min) | |  |  |  |  |  |  |  |  |
| Type of fuel | |  |  |  |  |  |  |  |  |
| Status of door and window | |  |  |  |  |  |  |  |  |
| Ventilation devices activated | |  |  |  |  |  |  |  |  |
|  | |  |  |  |  |  |  |  |  |
| Number of living room occupants throughout day and during burning period | |  |  |  |  |  |  |  |  |
| Any other sources of fumes (smokers, outdoor garbage burning, dust storm, etc.) | |  |  |  |  |  |  |  |  |
| Any other owner activity during the sampling: chemical cleaners, hoover, source (if there is any) | |  |  |  |  |  |  |  |  |
| Temperature (°C) and humidity (%) (indoors the sampling house) | |  |  |  |  |  |  |  |  |
| Other relevant comments and observations | |  |  |  |  |  |  |  |  |

**Table S3**. Descriptive statistics of Temperature and Relative humidity (RH) values obtained during the burning periods and non-burning periods in home operating improved stove (HO1, HO2, HO3, and HO4) and open fireplace (HO5) in 1-min time average (minimum values, 1^st^ quartile, median, 3^rd^ quartile, and maximum concentrations). BP refers to the ‘burning period’ and NBP to the ‘non-burning period’.

| **Period** | BP | BP | BP | BP | BP | NBP | NBP | NBP | NBP | NBP | Total | Total | Total | Total | Total |
| --- | --- | --- | --- | --- | --- | --- | --- | --- | --- | --- | --- | --- | --- | --- | --- |
| **Homes** | HO1 | HO2 | HO3 | HO4 | HO5 | HO1 | HO2 | HO3 | HO4 | HO5 | HO1 | HO2 | HO3 | HO4 | HO5 |
| **Temp_min (◦C)** | 18.3 | 15 | 17.9 | 16 | 14.2 | 17.3 | 14 | 16.7 | 15.8 | 13.2 | 17.3 | 14 | 16.7 | 15.8 | 13.2 |
| **Temp_median (◦C)** | 20.6 | 21.5 | 22.5 | 20.4 | 17.3 | 19.3 | 17.9 | 19.8 | 19.1 | 15.7 | 19.5 | 20.3 | 21.1 | 20 | 16 |
| **Temp_mean (◦C)** | 20.5 | 21.4 | 22.9 | 20.9 | 17. | 19.22143 | 17.77295 | 20.2 | 19.09 | 15.67 | 19.652 | 20.148 | 21.1027 | 20.118 | 16.19 |
| **Temp_**  **stdev (◦C)** | 0.964 | 1.5681 | 1.6243 | 1.774729 | 2.193342 | 0.755034 | 1.384014 | 1.728 | 1.376 | 1.35 | 1.0279 | 2.3137 | 2.02423 | 1.8473 | 1.839 |
| **Temp_q25 (◦C)** | 19.9 | 20.4 | 21.5 | 19.7 | 16.1 | 18.7 | 17 | 19 | 18 | 14.7 | 19 | 18.5 | 19.4 | 19 | 14.9 |
| **Temp_q75 (◦C)** | 21 | 22.6 | 23.6 | 22 | 19.3 | 19.7 | 18.8 | 21.6 | 20.1 | 16.5 | 20.3 | 21.9 | 22.7 | 21.1 | 17 |
| **Temp_max (◦C)** | 23.3 | 26.3 | 26.8 | 27.9 | 23.4 | 21.7 | 21.4 | 24.6 | 23 | 21.4 | 23.3 | 26.3 | 26.8 | 27.9 | 23.4 |
| **Humidity_min (%)** | 40.2 | 30.6 | 42 | 30.7 | 45.7 | 43.6 | 34.9 | 41.4 | 38.8 | 53 | 40.2 | 30.6 | 41.4 | 30.7 | 45.7 |
| **Humidity median (%)** | 48.4 | 39.7 | 47.6 | 42.7 | 57.9 | 49 | 41 | 49.1 | 44.7 | 61.3 | 48.9 | 40.3 | 48.5 | 43.4 | 60.4 |
| **Humidity_mean (%)** | 47.42 | 39.519 | 47.639 | 42.5374 | 57.33491 | 48.96712 | 41.31338 | 48.85 | 45.24 | 61.1 | 48.447 | 40.153 | 48.3737 | 43.708 | 60.12 |
| **Humidity_stdev (%)** | 3.012 | 3.812 | 2.264 | 2.783763 | 4.1875 | 2.135371 | 2.792076 | 2.164 | 2.91 | 3.556 | 2.5711 | 3.5896 | 2.28284 | 3.1388 | 4.081 |
| **Humidity_q25 (%)** | 45.2 | 36.4 | 46.2 | 41.2 | 54.7 | 47.4 | 39.2 | 47.2 | 43.2 | 58.2 | 46.7 | 37.7 | 46.6 | 42 | 57.2 |
| **Humidity_q75 (%)** | 49.8 | 42.5 | 49.3 | 44.2 | 59.9 | 50.7 | 43.6 | 50.4 | 47 | 64 | 50.2 | 42.9 | 50.1 | 45.2 | 63.2 |
| **Humidity_max(%)** | 52.7 | 55.4 | 53.2 | 51.8 | 67.8 | 55.6 | 50 | 61.1 | 52.9 | 68 | 55.6 | 55.4 | 61.1 | 52.9 | 68 |

**Table S4.** Descriptive statistics of PM_10_, PM_2.5_, PM_1_, BC, UFP, and CO concentrations obtained during the burning periods and non-burning periods in home operating improved stove (HO1, HO2, HO3, and HO4) and open fireplace (HO5) in 1-min time average (minimum values, 1^st^ quartile, median, 3^rd^ quartile, and maximum concentrations). * refers to missing UFP values at HO4 due to the Testo minidisc malfunctioning (19 Feb 2023 -02 March 2023). BP refers to the ‘burning period’ and NBP to the ‘non-burning period’.

| Period | BP | BP | BP | BP | BP | NBP | NBP | NBP | NBP | NBP | Total | Total | Total | Total | Total |
| --- | --- | --- | --- | --- | --- | --- | --- | --- | --- | --- | --- | --- | --- | --- | --- |
| HomeID | HO  1 | HO  2 | HO  3 | HO4 | HO5 | HO1 | HO2 | HO3 | HO4 | HO5 | HO1 | HO2 | HO3 | HO4 | HO5 |
| PM10_min (µg m^-3^) | 3.2 | 2.3 | 2.4 | 1 | 12 | 0.3 | 1.3 | 0.6 | 0.8 | 0.2 | 0.3 | 1.3 | 0.6 | 0.8 | 0.2 |
| PM10_med (µg m^-3^) | 20.3 | 37.9 | 24.5 | 14 | 89.6 | 6 | 11.4 | 7.1 | 3.4 | 8.4 | 8.9 | 23.7 | 11.6 | 5.6 | 14.4 |
| PM10_q25 (µg m^-3^) | 12.8 | 21.2 | 16 | 7.1 | 56.325 | 3.5 | 4.125 | 4.1 | 2.3 | 3.2 | 4.1 | 10.9 | 5.3 | 2.9 | 4.3 |
| PM10_q75 (µg m^-3^) | 34.5 | 67.1 | 37.9 | 28 | 145.35 | 12.4 | 20.2 | 15.5 | 5.9 | 21.4 | 16.6 | 47.7 | 23.6 | 13.2 | 43.3 |
| PM10_max (µg m^-3^) | 165.7 | 2962.1 | 634.9 | 1750.5 | 4117.8 | 571.7 | 281.4 | 112 | 75.3 | 360.1 | 571.7 | 2962.1 | 634.9 | 1750.5 | 4117.8 |
| P-value | 0.001 | 0.002 | 0.003 | 0.004 | 0.0002 | 0.001 | 0.001 | 0.001 | 0.001 | 0.0004 | 0.001 | 0.001 | 0.001 | 0.002 | 0.004 |
| PM2.5_min (µg m^-3^) | 1.4 | 2 | 1.8 | 1 | 5.6 | 0.2 | 1.3 | 0.6 | 0.8 | 0.2 | 0.2 | 1.3 | 0.6 | 0.8 | 0.2 |
| PM2.5_med (µg m^-3^) | 4.9 | 14.2 | 9.3 | 5.2 | 38.4 | 2.6 | 8 | 5.3 | 2.9 | 4.6 | 2.9 | 11.4 | 6.3 | 3.7 | 6.2 |
| PM2.5_q25 (µg m^-3^) | 3.4 | 8.4 | 6.1 | 3.9 | 22.3 | 1.6 | 3 | 3.4 | 2.1 | 2.5 | 1.9 | 6 | 4 | 2.5 | 3.1 |
| PM2.5_q75 (µg m^-3^) | 7.6 | 25.3 | 13.8 | 7.2 | 87.8 | 3.7 | 14.6 | 8.7 | 4.2 | 7.8 | 4.5 | 21.7 | 11 | 5.5 | 14.6 |
| PM2.5_max (µg m^-3^) | 87.7 | 344.5 | 104.4 | 100.8 | 3344.8 | 361.7 | 40.5 | 44.5 | 24.6 | 152.3 | 361.7 | 344.5 | 104.4 | 100.8 | 3344.8 |
| PM1_min (µg m^-3^) | 0.6 | 1.2 | 0.7 | 0.8 | 1.9 | 0.1 | 1.1 | 0.6 | 0.7 | 0.2 | 0.1 | 1.1 | 0.6 | 0.7 | 0.2 |
| PM1_med (µg m^-3^) | 2.4 | 10 | 5.2 | 2.5 | 28.55 | 1 | 6.3 | 4.2 | 2.2 | 2.7 | 1.2 | 8.3 | 4.6 | 2.4 | 3.5 |
| PM1_q25  (µg m^-3^) | 1.5 | 5.1 | 3.2 | 2 | 13.2 | 0.6 | 2.1 | 2.5 | 1.6 | 1.5 | 0.7 | 3.5 | 2.8 | 1.8 | 1.8 |
| PM1_q75  (µg m^-3^) | 4.7 | 21 | 9.2 | 3.5 | 79.6 | 1.5 | 13.6 | 6.8 | 3 | 4.4 | 1.9 | 18.4 | 7.3 | 3.2 | 8.8 |
| PM1_max  (µg m^-3^) | 72.6 | 325.7 | 88.9 | 53.1 | 2123.2 | 167.7 | 31.8 | 23.9 | 21.8 | 58.7 | 167.7 | 325.7 | 88.9 | 53.1 | 2123.2 |
| BC_min  (µg m^-3^) | 0.001 | 0.002 | 0.002 | 0.002 | 0.013 | 0 | 0 | 0 | 0.001 | 0 | 0 | 0 | 0 | 0.001 | 0 |
| BC_med  (µg m^-3^) | 0.48 | 1.47 | 0.4 | 0.59 | 1.70 | 0.25 | 0.47 | 0.36 | 0.39 | 0.23 | 0.30 | 1.04 | 0.4 | 0.47 | 0.35 |
| BC_q25  (µg m^-3^) | 0.25 | 0.71 | 0.35 | 0.36 | 0.82 | 0.12 | 0.24 | 0.19 | 0.25 | 0.12 | 0.14 | 0.45 | 0.23 | 0.30 | 0.16 |
| BC_q75  (µg m^-3^) | 0.95 | 3.02 | 0.79 | 0.83 | 4.41 | 0.52 | 0.998 | 0.54 | 0.55 | 0.42 | 0.664 | 2.40 | 0.62 | 0.70 | 0 |
| BC_max  (µg m^-3^) | 5.46 | 165.09 | 4.49 | 5.70 | 354.73 | 21.46 | 8.70 | 2.58 | 2.11 | 3.68 | 21.46 | 165.09 | 4.49 | 5.70 | 354.73 |
| PNC_min  (# cm^-3^) | 1258 | 1582 | 1885 | * | 1241 | 30 | 216 | 525 | * | 143 | 30 | 216 | 525 | * | 143 |
| PNC_med  (# cm^-3^) | 16877 | 21851 | 13614 | * | 36264.5 | 2053.5 | 3940 | 2481 | * | 3207.5 | 3390 | 11247 | 3474.5 | * | 4836 |
| PNC_q25  (# cm^-3^) | 7894.75 | 10867 | 8002 | * | 15376.25 | 699 | 2467.5 | 1645 | * | 1579 | 846 | 5054 | 1908 | * | 1940 |
| PNC_q75  (# cm^-3^) | 39420 | 59858.5 | 26158 | * | 73037.5 | 7861 | 6037 | 4354 | * | 5545.5 | 14775.5 | 34962 | 8334 | * | 16406 |
| PNC_max  (# cm^-3^) | 1013146 | 1047245 | 154700 | * | 3540433 | 417629 | 107669 | 99064 | * | 241443 | 1013146 | 1047245 | 154700 | * | 3540433 |
| CO_min  (ppm) | 0.5 | 0.4 | 0.5 | 0.4 | 0.1 | 0.5 | 0.1 | 0.4 | 0.4 | 0.1 | 0.5 | 0.1 | 0.4 | 0.4 | 0.1 |
| CO_med  (ppm) | 0.6 | 0.8 | 0.9 | 0.6 | 1.3 | 0.6 | 0.6 | 0.6 | 0.6 | 0.1 | 0.6 | 0.6 | 0.7 | 0.6 | 0.1 |
| CO_q25  (ppm) | 0.6 | 0.6 | 0.7 | 0.6 | 0.8 | 0.6 | 0.5 | 0.6 | 0.6 | 0.1 | 0.6 | 0.6 | 0.6 | 0.6 | 0.1 |
| CO_q75  (ppm) | 0.9 | 1.3 | 1.3 | 0.8 | 2.8 | 0.6 | 0.6 | 0.8 | 0.6 | 0.2 | 0.6 | 1 | 1 | 0.7 | 0.4 |
| CO_max  (ppm) | 2.1 | 6.7 | 1.7 | 1.5 | 27.6 | 1 | 3.1 | 2.2 | 1.1 | 1 | 2.1 | 6.7 | 2.2 | 1.5 | 27.6 |

Table S5.Home plan and detailed description of the studied homes.


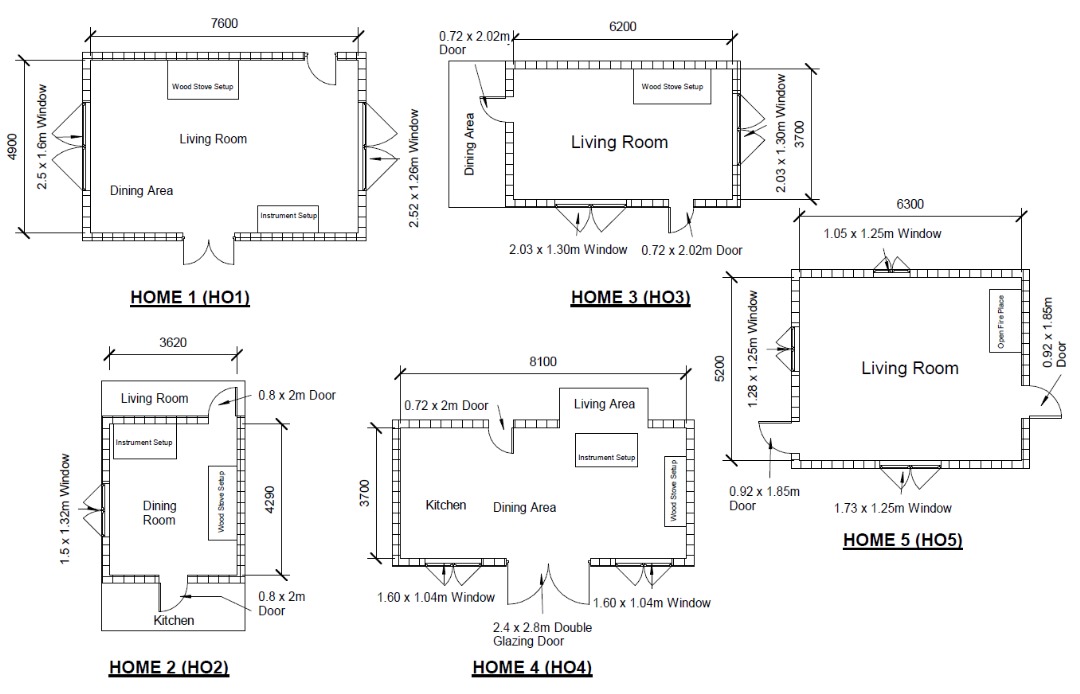


| **Detailed Description of the studied homes – see home plans above** |
| --- |
| The choice of monitored homes was based on the knowledge of the researchers to ensure the selected homes meet the criteria specified in Section 4.1. Wood-burning stove across all homes were installed on the ground floor. Four fuel types (KD, SW, SC and WB) were used in four homes (HO2, HO3, HO4 and HO5), except in HO1 with an eco-design stove that does not support burning of SC. Detailed characteristics of each home are as follows:   - Home 1 (HO1) is located at the East Shalford Village, Guildford. A 4-bedroom detached house in a relatively low polluted and less congested area, surrounded by trees. HO1 was made of bricks, wood, and cement, though an old building. The living room (7.6m×4.9m×2.3m) has three windows (2.52m×1.26m; 2.5m×1.6m; 1.5.5m×0.73m), and was situated next to the kitchen area. The living room has a wooden floor with a central carpet. The home has oil based central heating as a main source of heating during winter and uses a wood stove as a secondary heating source. The Eco stove was placed in the living area and operated majorly in the evening to burn only wood from around 4 pm till midnight, except on weekends. - Home 2 (HO2) is situated in the semi-rural, Normandy area of Guildford, 5m away from a moderate traffic road. This semi-detached house has 3-bedrooms and 2 reception rooms. The ground floor has two separate reception rooms, two bedrooms on the first floor, and a third bedroom on the second floor. The house accommodated a minimum of 4 occupants. The multifuel eco-design stove is situated in the dining area (4.29m×3.62m×2.5m) that has wooden floor area with one double glazing window (1.5m x1.32m) and a door (2m x 0.8m) opening to the living room and another to the kitchen. The home has a gas central heating for the bedrooms but uses the stove as a primary heating source for the ground floor only. The stove was operated all day until midnight, and the fire restarted again during the morning hours. - Home 3 (HO3) is situated – at Merrow area of Guildford, 10 m away from a major road. A four-bedroom detached house. The small multifuel Eco-design stove is in the living room with a wooden floor slab and a centre carpet. The living room (6.2m×3.7m×2.5m) has 2 double glazing windows (each 2.03m×1.30m) and 2 doors (each 2.02m×0.72m) openable outside, and the dining area. The home has electric central heating as a main source of heating during winter and uses a wood stove as a secondary heating source in the living area and operated majorly in the evening from 16:00h till midnight, except on weekends. - Home 4 (HO4) is situated at Onslow area of Guildford. This detached 3-bedroom house is spread over 1819 square feet, bounded at the rear 20m away from a heavy traffic A3 road. It has a clear skies stage (v) stove, which is the latest improved stove approved by DEFRA (2022 model). The home is made of bricks and cement, accommodating a minimum of four people, with the stove located in a combined dining/kitchen room. The kitchen and the wood-burning stove are situated in the same room without the use of extraction fans during cooking throughout the monitoring period. The Testo minidisc instrument was beeping at a high concentration from the first day of monitoring in this home, and it got clogged and was taken for repair due to exposure to the very high concentration of UFP in this home, and the UFP data was not collected in this home. The dining area has two double-glazed windows of dimension (1.60m × 1.04m), and three rooftop windows (0.6m × 1m), which are always closed but have sliding double glass doors (2.4m×2.8m) that are open to the A3 road. - Home 5 (HO5) is located at the Bramley Village, Nurscombe Farm Cottage House. It is a house of historical interest, an old castle built over 400 years ago with 5 bedrooms. The home is equipped with a traditional open fireplace operated manually in batch mode, and with no control of combustion air. The fireplace is characterized by a combustion chamber with a volume of 0.09 m^3^, corresponding to 0.35 m height, with a trapezoidal base of 0.52 and 0.63 m width and 0.45 m depth. The living room (5.2m×6.3m×2.7m) has a wooden floor with a central carpet, with three windows (1.73m×1.25m; 1.28m×1.25m; 1.05m×1.25m), and 2 doors (each 1.85m×0.92m) openable outside. The home has an electric central heating as a main source of heating during winter for bedrooms and kitchen, but uses a wood stove as a secondary heating source for the living room only. The open fire place is operated majorly in the evening from around 4 pm till midnight, except on weekends.   HO1, HO3 and HO4 used the heating stove as a primary source of heating in the living area. Other activities in all the five homes during the monitoring periods included cooking, sweeping, and vacuuming. The amount of burning time was significantly lower in HO5 than HO2 because the wood burner is only used as a secondary source of heating. All the homes are naturally ventilated with openable windows and doors. HO2 and HO3 were located on a busy road. All the home is naturally ventilated without any mechanical ventilation system. Also, because of winter periods, the door and window are shut to keep the house warm, except for HO1 which opens the windows sometimes during cooking. |

Figure S1. Time variation of CO concentration (ppm) measured during the monitoring periods in Home 1 (improved woodstove)

Figure S2. Time variation of CO concentration (ppm) measured during the monitoring periods in Home 2 (improved multifuel woodstove)

Figure S3. Time variation of CO concentration (ppm) measured during the monitoring periods in Home 3

Figure S4. Time variation of CO concentration (ppm) measured during the monitoring periods in Home 4

Figure S5. Time variation of CO concentration (ppm) measured during the monitoring periods in Home 5 (open fireplace).

Figure S6. Time variation of UFP concentration (# cm^-3^) measured during the monitoring periods in Home1.

Figure S7. Time variation of UFP concentration (# cm^-3^) measured during the monitoring periods in Home2

Figure S8. Time variation of UFP concentration (# cm^-3^) measured during the monitoring periods in Home3

Figure S9. Time variation of UFP concentration (# cm^-3^) measured during the monitoring periods in Home 5.

Figure S10. Time series of UFP concentration (# cm^-3^) measured during the monitoring periods in Home 2.

Figure S11. Time variation of PM_10_, PM_2.5_, and PM_1_ concentration during the monitoring periods in Home 1

Figure S12. Time variation of PM_10_, PM_2.5_, and PM_1_ concentration (µgm^-3^) measured during the monitoring periods in Home2

Figure S13. Time variation of PM_10_, PM_2.5_, and PM_1_ concentration (µgm^-3^) measured during the monitoring periods in Home 3.

Figure S14. Time variation of PM_10_, PM_2.5_, and PM_1_ concentration (µgm^-3^) measured during the monitoring periods in Home 4.

Figure S15. Time variation of PM_10_, PM_2.5_, and PM_1_ concentration (µgm^-3^) measured during the monitoring periods in Home 5.

**Figure S16.** Images of improved heat stoves used in the experiments, and instrument setup. (a) Eco stoves; (b-c) multifuel stove; (d) Clear skies stage 5 stoves; (e) open fireplace; (f-g) instrument experimental setup inside the casing for Indoor Air Quality monitoring.

**References**

1 Martins, N. R. & Carrilho da Graça, G. Health effects of PM2.5 emissions from woodstoves and fireplaces in living spaces. *Journal of Building Engineering* **79**, 107848 (2023).

2 Feliciano, M., Lira, F., Furst, L. C. & Arioli, R. The influence of domestic heating systems in indoor air quality in homes of a region of Northeastern Portugal. *Progress in Industrial Ecology, an International Journal* **15**, 162-182 (2022).

3 Rahman, M. *et al.* Cleaning the Flue in Wood-Burning Stoves Is a Key Factor in Reducing Household Air Pollution. *Toxics* **10**, 615 (2022).

4 Walker, E. S. *et al.* Indoor fine particulate matter and demographic, household, and wood stove characteristics among rural US homes heated with wood fuel. *Indoor air* **31**, 1109-1124 (2021).

5 Chakraborty, R., Heydon, J., Mayfield, M. & Mihaylova, L. Indoor Air Pollution from Residential Stoves: Examining the Flooding of Particulate Matter into Homes during Real-World Use. *Atmosphere* **11**, 1326 (2020).

6 Fleisch, A. F. *et al.* Residential wood stove use and indoor exposure to PM2. 5 and its components in Northern New England. *Journal of exposure science & environmental epidemiology* **30**, 350-361 (2020).

7 Vicente, E. D. *et al.* Impact of wood combustion on indoor air quality. *Science of The Total Environment* **705**, 135769 (2020).

8 Hamon, M. *et al.* in *Cold Climate HVAC 2018.* (eds Dennis Johansson, Hans Bagge, & Åsa Wahlström) 887-897 (Springer International Publishing).

9 Frasca, D. *et al.* Influence of advanced wood-fired appliances for residential heating on indoor air quality. *Chemosphere* **211**, 62-71 (2018).

10 De Gennaro, G. *et al.* Discontinuous and continuous indoor air quality monitoring in homes with fireplaces or wood stoves as heating system. *International journal of environmental research and public health* **13**, 78 (2016).

11 Wyss, A. B. *et al.* Particulate matter 2.5 exposure and self-reported use of wood stoves and other indoor combustion sources in urban nonsmoking homes in Norway. *PloS one* **11**, e0166440 (2016).

12 Semmens, E. O., Noonan, C. W., Allen, R. W., Weiler, E. C. & Ward, T. J. Indoor particulate matter in rural, wood stove heated homes. *Environmental Research* **138**, 93-100 (2015).

13 Canha, N. *et al.* Impact of wood burning on indoor PM2.5 in a primary school in rural Portugal. *Atmospheric Environment* **94**, 663-670 (2014).

14 Salthammer, T., Schripp, T., Wientzek, S. & Wensing, M. Impact of operating wood-burning fireplace ovens on indoor air quality. *Chemosphere* **103**, 205-211 (2014).

15 Carvalho, R. L., Jensen, O. M., Afshari, A. & Bergsøe, N. C. Wood-burning stoves in low-carbon dwellings. *Energy and Buildings* **59**, 244-251 (2013).

16 Allen, R. W., Leckie, S., Millar, G. & Brauer, M. The impact of wood stove technology upgrades on indoor residential air quality. *Atmospheric Environment* **43**, 5908-5915 (2009).

17 Ward, T., Palmer, C., Bergauff, M., Hooper, K. & Noonan, C. Results of a residential indoor PM2. 5 sampling program before and after a woodstove changeout. *Indoor air* **18**, 408-415 (2008).

18 Guo, L., Lewis, J. & McLaughlin, J. Emissions from Irish domestic fireplaces and their impact on indoor air quality when used as supplementary heating source. *Global NEST J* **10**, 20 (2008).

19 Gustafson, P., Östman, C. & Sällsten, G. Indoor levels of polycyclic aromatic hydrocarbons in homes with or without wood burning for heating. *Environmental Science & Technology* **42**, 5074-5080 (2008).

20 Glasius, M. *et al.* Impact of wood combustion on particle levels in a residential area in Denmark. *Atmospheric Environment* **40**, 7115-7124 (2006).
